# Inflammasome Priming Mediated *via* Toll-Like Receptors 2 and 4, Induces Th1-Like Regulatory T Cells in *De Novo* Autoimmune Hepatitis

**DOI:** 10.3389/fimmu.2018.01612

**Published:** 2018-07-19

**Authors:** Adam S. Arterbery, Jie Yao, Andrew Ling, Yaron Avitzur, Mercedes Martinez, Steven Lobritto, Yanhong Deng, Gan Geliang, Sameet Mehta, Guilin Wang, James Knight, Udeme D. Ekong

**Affiliations:** ^1^Pediatric Gastroenterology and Hepatology, Yale University, New Haven, CT, United States; ^2^Gastroenterology, Hepatology, and Nutrition, Hospital for Sick Children, Toronto, ON, Canada; ^3^Pediatric Gastroenterology, Hepatology, and Nutrition, Columbia University, New York, NY, United States; ^4^Yale Center for Analytical Sciences, New Haven, CT, United States; ^5^Yale Center for Genome Analysis, Yale School of Medicine, New Haven, CT, United States

**Keywords:** innate immunity, toll-like receptors, inflammasome, Th1 regulatory T cells, liver transplantation, CD14^++^ monocytes, autoimmune hepatitis, tumor necrosis factor α-induced protein 3

## Abstract

*De novo* autoimmune hepatitis (DAIH) is an important cause of late allograft dysfunction following liver transplantation, but its cause and underlying pathogenesis remains unclear. We sought to identify specific innate and adaptive immune mechanisms driving the pro-inflammatory cytokine secreting regulatory T cell (Treg) phenotype in DAIH and determine if modulation of these pathways could resolve the inflammatory milieu observed in the livers of patients with DAIH. Here, we demonstrate toll-like receptors (TLRs) 2- and 4-mediated inflammasome activation in CD14^++^ monocytes, a finding that is key to maintaining dysfunctional Tregs in patients with DAIH. Furthermore, silencing of TLR 2 and 4 in CD14^++^ monocytes prevented activation of the inflammasome and significantly decreased IFN-γ production by FOXP3^+^ Tregs. We also observed significantly increase in expression of tumor necrosis factor α-induced protein 3 (*TNFAIP3*), a negative regulator of the NLRP3 Inflammasome, in monocytes/macrophages of liver transplant subjects who have normal allograft function and do not have DAIH. *TNFAIP3* expression was virtually absent in monocytes/macrophages of patients with DAIH. Our findings suggest that autoimmunity in DAIH is promoted by CD14^++^ monocytes predominantly through activation of inflammatory signaling pathways.

## Introduction

*De novo* autoimmune hepatitis (DAIH) is an important cause of late allograft dysfunction after liver transplantation, but its cause and underlying pathogenesis remains unclear. We have previously reported that CD14^++^monocyte-derived IL-12, directs regulatory T cells (Tregs) to differentiate into dysfunctional, IFN-γ secreting, Th1-like effector Tregs (1). Furthermore, we observed that these monocytes as well as CD68^+^ macrophages in the liver constitutively produce IL-1β (1). To better understand the mechanisms driving CD14^++^monocyte activation and Treg differentiation, we sought to identify specific innate and adaptive immune mechanisms that drive a pro-inflammatory, cytokine secreting, effector Treg phenotype. In addition, we examined whether modulation of these pathways could resolve the inflammatory milieu observed in the livers of patients with DAIH. Given that IL-1β is a signature downstream cytokine of inflammasome activation (2, 3), we hypothesized that activation of the innate immune system is the driver for the adaptive system in DAIH and that the chronic inflammation in DAIH is augmented by inflammasomes. Our results show that priming of the inflammasome mediated *via* toll-like receptors (TLRs) 2 and 4 is increased in DAIH and autoimmune hepatitis (AIH) and is key to maintaining dysfunctional Tregs. Our findings provide further insights into the balance and communication between innate and adaptive immune responses in DAIH that may lead to potential therapeutic options.

## Materials and Methods

### Study Design

This was a cross sectional study involving pediatric liver transplant recipients with a diagnosis of DAIH (*n* = 11), pediatric liver transplant recipients who had never been diagnosed with DAIH and had normal allograft function at the time of enrollment and blood draw. These subjects acted as the liver transplant control group (LTC) (*n* = 42), and healthy children who had no underlying immune-mediated disorders and were not on any immunomodulatory agents. These subjects acted as the healthy control group (HC) (*n* = 39). Finally, non-transplanted children with a diagnosis of AIH were also included (*n* = 9). The transplanted subjects were enrolled as they attended their routine post-transplant clinics, the non-transplanted children with AIH were enrolled as they attended their routine pediatric hepatology clinics, and the healthy non-transplanted subjects were enrolled using flyers posted in New Haven as well as from the *help us discover* database, a database of healthy children registered as research controls maintained by the Yale Center for Clinical Investigation. The definition of DAIH was as previously described (4): (a liver transplant recipient without a history of autoimmune liver disease presenting with unknown etiology of late graft dysfunction. Late graft dysfunction characterized by elevated aminotransferases, and graft dysfunction not due to any of the following causes: acute and chronic rejection, hepatitis B and C infection, Epstein Barr virus and Cytomegalovirus infections, vascular problems, biliary complication, drug toxicity, sepsis, recurrence of primary disease, or post-transplant lymphoproliferative disease; elevated serum immunoglobulin G, positive autoantibody titers: ANA, ASMA, anti-LKM; characteristic biopsy findings of dense lymphocytic portal tract infiltrate with plasma cells, and interface hepatitis) (4). The definition of AIH was as previously described (5, 6). Inclusion/exclusion criteria were established prospectively.

The inclusion criteria for the liver transplant control group were age ≤20 years, serum aminotransferases and gamma glutamyl transpeptidase ≤ULN at blood draw, no past diagnosis of DAIH, no biliary or vascular complication at time of blood draw, no past or present history of chronic rejection, no recent acute rejection (>1 year). The exclusion criteria included age ≥21 years, biliary/vascular complication at time of blood draw, serum aminotransferases and gamma glutamyl transpeptidase >ULN at blood draw, recent acute rejection (≤1 year), and history of chronic rejection. We enrolled patients aged 0 through 20 years from January 2015 to December 2017. The rule for stopping patient enrollment and sample collection was determined by the dates as stated above. At enrollment, 30 mL of peripheral blood was obtained from each participant, and every 3 months over the duration of the study. If the study participant underwent a clinically indicated liver biopsy, liver tissue was obtained for isolation of intrahepatic lymphocytes. Each experiment described was replicated twice with substantiation of the results. Informed consent was obtained from parents or guardians and assent was obtained as necessary per institutional review board guidelines. The study protocol was reviewed and approved by the Institutional Review Board of Yale University.

### Monocyte Stimulation Experiments

CD14^++^ monocytes were isolated, stimulated, stained, and subjected to FACS analysis for IL-1β as previously described (1).

### Western Blotting

CD14^++^ monocytes were isolated and stimulated as previously reported (1), and protein was harvested using MPER solution with PhosSTOP according to the manufacturer’s instructions. Protein was harvested in M-PER protein extraction reagent supplemented with PhosSTOP phosphatase inhibitor tablets according to the manufacturer’s instructions. Protein concentrations were measured using the Bradford method and equal amounts of protein were analyzed by western blotting. For CD14^++^monocytes, the following inflammasome-associated antibodies were used: NLRP3, ASC, (Pro) IL-1β, and (Pro) Caspase 1. All bands were subjected to densitometry using ImageJ, and referenced against GAPDH.

### Quantification of mRNA Expression Levels by RT-PCR

qRT-PCR was carried out as previously reported (1). Briefly, RNA was isolated using Qiagen RNeasy Micro Kit (Qiagen), following manufacturer’s guidelines and converted to cDNA using qScript cDNA Supermix according to the manufacturer’s instructions. For quantification of damage-associated molecular patterns (DAMPs), DNA was harvested from 200 μL of patient sera using QIAamp DNA Blood Mini Kit according to the manufacturer’s instructions. The following were targeted: DAMPs—*HMGB1, MT-ATP6, ACTB, Fibrinogen, HSP60, HSP70*, and *HSP90*. In addition, *pro-IL-1β* was quantified in CD14^++^ monocytes stimulated with lipopolysaccharide (LPS) (1 mg/mL) for 24 h. Probes and cDNA were mixed with SsoAdvanced Universal Probes Supermix and the reactions were set up following manufacturer’s guidelines and run on a 7500 Fast Real-Time PCR System (Applied Biosystems). Values are represented as the difference in cycle threshold values normalized to reference gene (β2M or 18s) for each sample as per the following formula: relative RNA expression = (2^−dCt^) × 1,000.

### Luminex—ELISA

Frozen plasma samples were thawed completely and centrifuged for 5 min prior to addition to the assay. Three Milliplex Map kits (EMD Millipore, Bilerica, MA, USA) were used to measure mitochondrial proteins in one assay (Complexes I–IV, Human Oxidative Phosphorylation Magnetic Bead Panel); heat shock proteins in another (Heat Shock Protein Magnetic Bead 5-Plex Kit); Fibrinogen protein in the third (Human Cardiovascular Disease Magnetic Bead Panel 3—Fibrinogen 1 Plex); and S100A8 and S100A12 in the fourth and fifth kit, respectively. Samples were incubated overnight at 4°C with a mixture of magnetic beads coupled to an antibody specific for one of the analytes. After washing the beads in the 96-well plates using a hand held magnet, the beads were incubated for 1 h at room temperature with biotinylated detection antibodies, washed, and then incubated for 30 min at room temperature with streptavidin–phycoerythrin. Plates were read in a Luminex 200 Analyzer (Luminex, Austin, TX, USA) controlled by xPONENT software. Values for each analyte were determined using Analyst software (EMD Millipore) from a standard curve of log dose vs. median fluorescent intensity using a five parameter logistic fit. All standards and samples were run in duplicate. The standard curve was used to calculate the concentration of QC controls supplied with the kits and found to be in the expected range.

### TLR Reporter Cell Lines and Sera Treatment (Inhibition)

Stimulation by proteins in patient sera: HEK cells that stably co-express a human TLR2, TLR4, or TLR9 gene and an NF-κB-inducible secreted embryonic alkaline phosphatase (SEAP) reporter gene were used to determine activation of TLRs from patient sera. Expression of SEAP under control of NF-κB/AP-1 promoters is inducible by TLR activation, and extracellular SEAP in the supernatant is proportional to NF-κB induction. Secreted SEAP levels quantified in the supernatant on the basis of optical density (colorimetric intensity) at 650 nm. Positive controls were as follows: TLR2—FSL1 (100 μg/mL), TLR4—LPS (100 ng/mL), and TLR9—OD2006 (100 μg/mL). Following the culture of TLR reporter cell lines according to the manufacturer’s instructions, 50 μL of sera (diluted 1:1 with PBS) was added and cultured for 24 h. The following day, 50 μL of supernatant was withdrawn from each culture, added to 200 μL Qunati-Blue, and incubated according to the manufacturer’s instructions. Colorimetric changes were observed and secreted SEAP levels quantified in the supernatant on the basis of optical density (colorimetric intensity) at 650 nm. Inhibition of proteins in patient sera: TLR reporter lines were cultured with patient sera as above. Protein inhibitors for Fibrinogen, HSPs (90), and Mitochondrial Complexes were applied to the cell cultures for 48 h at the following concentrations: 1 mm 17AAG (HSPs); 10 μM Fibrinogen Inhibitor (Fibrinogen); 25 μM Rotenone (Complex I); 10 mM TTFA (Complex II); 20 μM Antimycin A (Complex III). Colorimetric changes were observed and secreted SEAP levels quantified in the supernatant on the basis of optical density (colorimetric intensity) at 650 nm.

### Inhibition of TLRs in CD14^++^ Monocytes Using shRNA

Lentiviral particles expressing shRNAs were obtained from Origene (Rockville, MD, USA) for TLR2, TLR4, and TLR9. CD14^++^ monocytes were subject to the recommended manufacturer’s protocol for transduction of non-adherent primary cells. In brief, CD14^++^ monocytes were obtained by positive selection (18058; STEMCELL) from peripheral blood mononuclear cells (PBMCs) following manufacturer’s guidelines. For transduction, 5 × 10^4^ human CD14^++^ monocytes were transduced with viral particles containing a vector expressing TLR-specific shRNA or as controls a vector expressing an unspecific shRNA. Transduction was mediated at a multiplicity of infection of 5 by centrifugation at 2,250 rpm for 30 min at room temperature in the presence of 3 μg/mL polybrene (Millipore, Billerica, MA, USA). Cells were then cultured in Xvivo15 media with LPS (1 mg/mL) for 24 h at 37°C with 5% CO_2_. The following day, cells were gently re-suspended and one-third the volume was harvested for RNA, converted to cDNA, and subjected to qRT-PCR as described above; and analyzed for the expression of TR2, TLR4, and TLR9, respectively. Following the confirmation of knockdown by qRT-PCR, 10,000 transduced CD14^++^ monocytes were then co-cultured with 50,000 CD4^+^CD25^hi^CD127^−^ Tregs for 5 days as previously described (1). Cells were then subjected to intracellular staining of cytokine, Tregs were analyzed for expression of FOXP3 and IFN-γ expression, and CD14^++^monocytes were analyzed for IL-6, IL-12, and IL-1β expression using flow cytometry as previously described (1); or cells were harvested for RNA for analysis of TLR gene expression by qPCR and confirmation of transient knockdown. In addition, CD14^++^ monocytes were analyzed by western blot for inflammasome-associated proteins as described above.

### Single-Cell Sequencing

CD14^++^ monocytes from PBMCs and CD68^+^ macrophages from human liver were FACS sorted for use in single-cell sequencing. Briefly, sorted cells were counted and assessed for viability with Trypan Blue using a Countess II automated counter (Invitrogen), and adjusted at a concentration of 800–1,000 cells/μL. Final cell viability estimates ranged between 85 and 95%. To generate single-cell gel beads in emulsion (GEMs) and sequencing libraries, single-cell suspensions were loaded onto 10X Genomics Single Cell A Chips (10x Genomics, PN-120236) along with the reverse transcription master mix as per the manufacturer’s protocol for the Chromium Single Cell 3′ Reagent kits V2 (10X Genomics; PN-120237). Libraries were sequenced on an Illumina HiSeq2500 using rapid mode as follows: 26 bp (Read 1); 8 bp (i7 Sample Index); 0 bp (i5 Index); and 98 bp (Reads 2). The Cell Ranger Single Cell Software Suite 2.1.0 by 10X Genomics (http://10xgenomics.com/) was used to process sequenced data into transcript count tables. Downstream processing and visualization was done using R and Seurat.

### Hepatocyte and CD14^++^ Monocyte Co-Culture

In brief, CD14^++^ monocytes were obtained by positive selection from PBMCs following manufacturer’s guidelines from subjects with DAIH, liver transplanted control (LTC) subjects and healthy, non-transplanted subjects. CD14^++^ monocytes were then co-cultured with normal human hepatocytes (obtained through the Liver Tissue Cell Distribution System, University of Pittsburgh, PA, USA) in a 1:4 ratio (monocyte:hepatocyte), for 24, 72, and 96 h at 37°C with 5% CO_2_; Cultured cells were then subjected to confocal microscopy for detection of anti-active caspase-3, and anti-CD14 as follows: cultured cells rinsed with PBS and fixed with methanol for 2 h on ice, subsequently incubated with the primary antibody overnight at 4°C, and secondary antibody over 2 h at room temperature. Primary and secondary antibodies were anti-human albumin antibody with Alexa Fluor 488-labeled IgG, anti-caspase-3 antibody with Alexa Fluor 555-labeled IgG, and anti-CD14 antibody with Alexa Fluor 647-labeled IgG, respectively. Imaging performed using a Leica YSCC SP5 confocal system at 100× objective. Culture supernatant was obtained for measurement of alanine aminotransferase. Measurement of alanine aminotransferase was performed using the Alfa Wassermann automated chemistry analyzer (Alera model).

## Results

### Demographics

Table 1 highlights the demographics of all enrolled subjects. For the transplanted subjects, six were on Sirolimus (two subjects with DAIH and four LTC subjects), and four on Cyclosporine (two subjects each in the DAIH and LTC group, respectively). The remaining transplanted subjects were all on Tacrolimus. Importantly there was no significant difference in levels of anti-rejection drugs and pre-transplant diagnosis between the two liver transplanted subject groups.

**Table 1:** Demographics.

	DAIH (*n* = 11)	LTC (*n* = 42)	HC (*n* = 39)	AIH (*n* = 9)	*p* Value
Median (range) age at blood draw (years)	16.1 (10.6–19.1)	7.4 (0.9–20.8)	7.6 (1.2–17.7)	9.5 (6.6–16.3)	*0.049
					^#^0.41
Median (range) duration from transplant at blood draw (years)	10.7 (1.9–19.3)	3.8 (0.1–19.6)			0.007
Mean ± SD	5.03 ± 1.1	4.93 ± 2.8	n/a	n/a	0.59
FK level at blood draw (ng/ml)					
Mean ± SD	86 ± 16.9	38.5 ± 27.5	n/a	n/a	0.25
CYA level at blood draw (ng/ml)					
Mean ± SD	1.65 ± 0.78	5.33 ± 3.44	n/a	n/a	0.25
Sirolimus level at blood draw (ng/ml)					
Gender (M/F)	4/7	27/15	20/19	2/7	*0.17
					^#^0.15
Graft type (DD/LD)	10/1	23/19	n/a	n/a	0.037
Pre-TX diagnosis					
Biliary Atresia	5	27	n/a	n/a	^$^0.31
Metabolic/genetic	1	10			^@^0.42
Other	5	5			

*DAIH vs. LTC.

*^#^AIH vs. HC*.

*^$,@^Proportion of either Biliary Atresia or metabolic/genetic disease is not significantly different between DAIH vs. LTC*.

*KEY*.

*DAIH, de novo autoimmune hepatitis; LTC, liver transplanted control; HC, healthy control; AIH, autoimmune hepatitis; DD, deceased donor; LD, living donor; FK, tacrolimus; CYA, cyclosporin*.

### Inflammasome Activation Occurs in CD14^++^ Monocytes From Patients With DAIH

**Figure 1:**
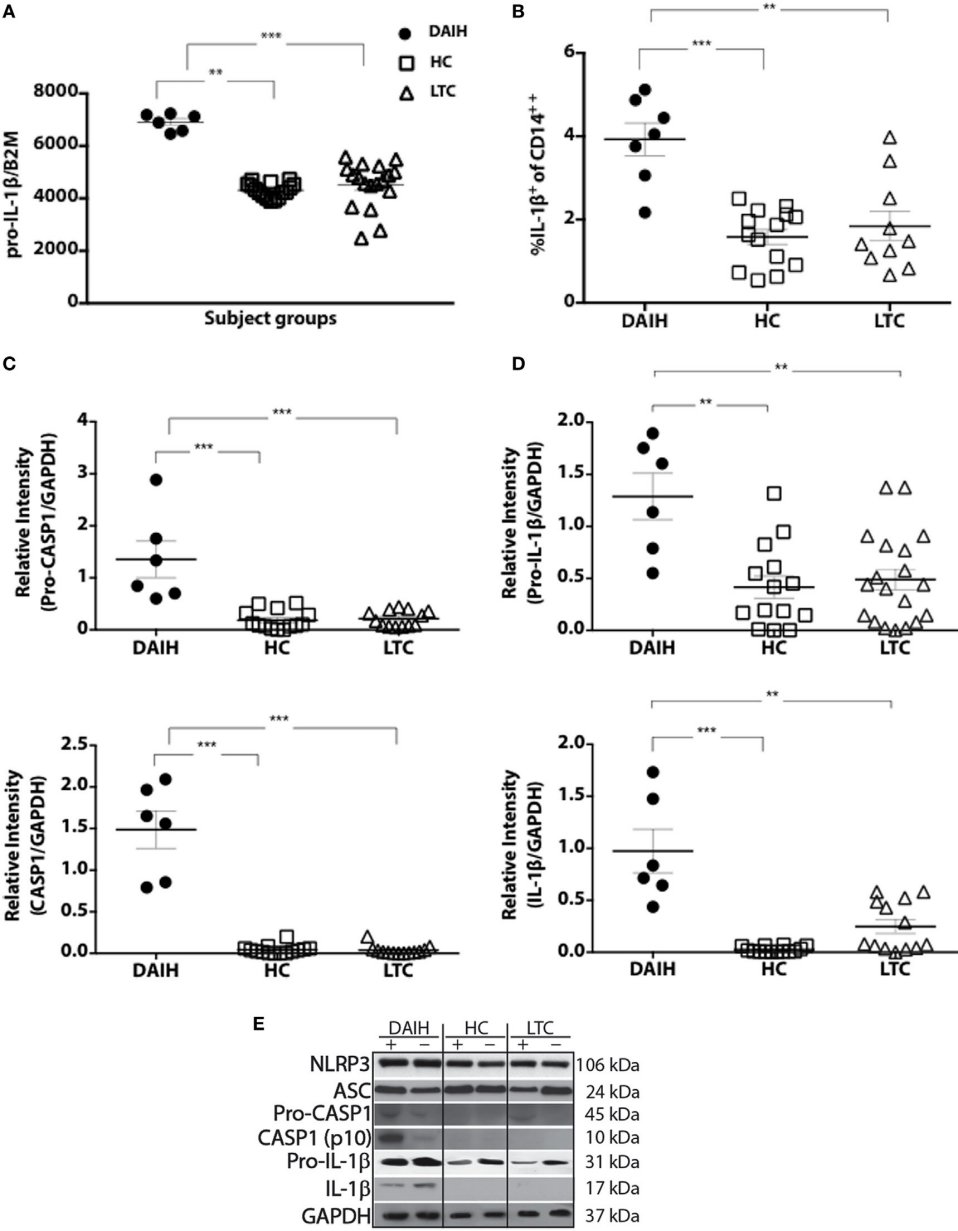
CD14^++^ monocytes from liver transplanted patients with *de novo* autoimmune hepatitis (DAIH) undergo inflammasome activation. CD14^++^ monocytes from liver transplanted patients with DAIH (*n* = 7) and without DAIH (LTC) (*n* = 18) and healthy, non-transplanted children (HC) (*n* = 16) were stimulated with lipopolysaccharide (LPS) for 24 h and (i) subjected to qPCR for pro-IL-1β; (ii) stained with anti-CD3, anti-CD14, intracellular IL-1β. Cytokine secretion analyzed using flow cytometry; (iii) subjected to Western Blot for NLRP3, ASC, pro-caspase-1, caspase-1, IL-1β. **(A)** CD14^++^ monocytes from patients with DAIH expressed significantly more *pro-IL-1β* compared to CD14^++^ monocytes from liver transplanted patients with normal allograft function and no DAIH (LTC) (*p* < 0.001), as well as CD14^++^ monocytes from healthy, non-transplanted children (HC) (*p* = 0.002). **(B)** CD14^++^ monocytes from patients with DAIH produced significantly more IL-1β compared to CD14^++^ monocytes from liver transplanted patients with normal allograft function and no DAIH (LTC) (*p* = 0.005), as well as CD14^++^ monocytes from healthy, non-transplanted children (HC) (*p* < 0.001). **(C–E)** Significant pro-caspase and caspase-1 cleavage observed in CD14^++^ monocytes from patients with DAIH compared to CD14^++^ monocytes from LTC (*p* < 0.001; *p* < 0.001) and HC (*p* < 0.001; *p* < 0.001). Significant pro-IL-1β and IL-1β production observed in CD14^++^ monocytes from patients with DAIH compared to CD14^++^ monocytes from LTC (*p* = 0.007; *p* = 0.002) and HC (*p* = 0.006; *p* < 0.001). Representative blot for three patients from each subject group. Plus sign indicates with LPS stimulation, minus sign indicates absence of LPS stimulation.

Given our published observation of constitutive IL-1β production in CD14^++^ monocytes of patients with DAIH (1), we investigated for inflammasome activation in CD14^++^ monocytes. CD14^++^monocytes from subjects with DAIH produced significantly more IL-1β and expressed significantly more *pro-IL-1β* compared to CD14^++^monocytes from liver transplanted patients with normal allograft function who did not have DAIH (LTC) and CD14^++^ monocytes from healthy, non-transplanted children (HC) (Figures 1A,B) (gating strategy Figure S1A in Supplementary Material).

We also observed significantly increased expression of inflammasome-associated effector proteins from CD14^++^ monocytes of patients with DAIH compared to expression in CD14^++^ monocytes from LTC and HC (Figures 1C,D). Together these results demonstrate increased caspase-1 cleavage, and expression of the downstream signature cytokine of inflammasome activation, IL-1β in CD14^++^ monocytes of patients with DAIH. Of note, we also observed similar caspase-1 cleavage with IL-1β secretion from CD14^++^ monocytes of non-transplanted subjects with AIH compared to CD14^++^ monocytes of healthy, non-transplanted children (Figure S1B in Supplementary Material). Thus, pathologic inflammasome activation in CD14^++^ monocytes appears to be a common feature in at least two forms of autoimmune liver disease.

### Inflammasome Activation Mediated by TLRs 2/4 and 9 in DAIH

**Figure 2:**
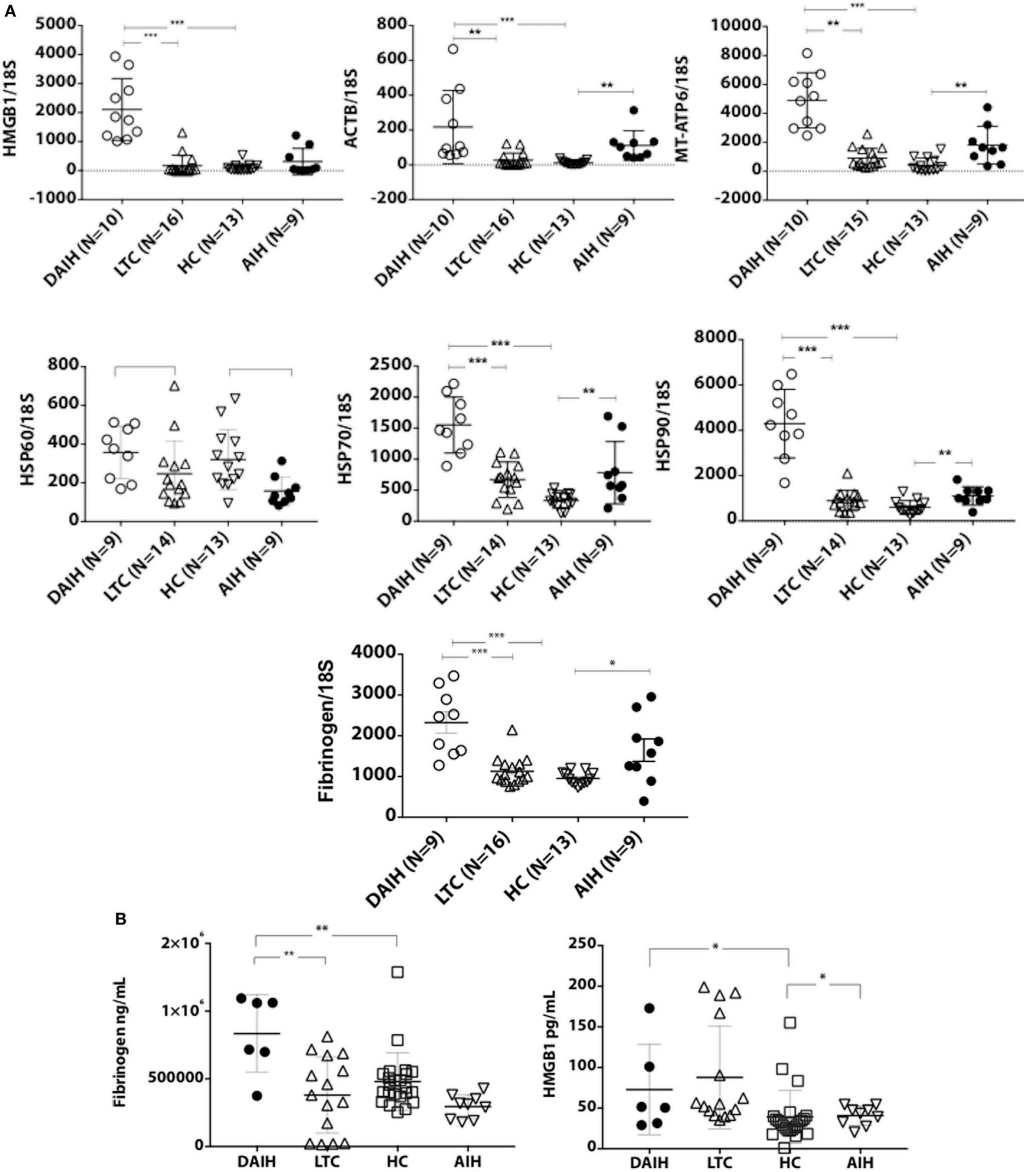

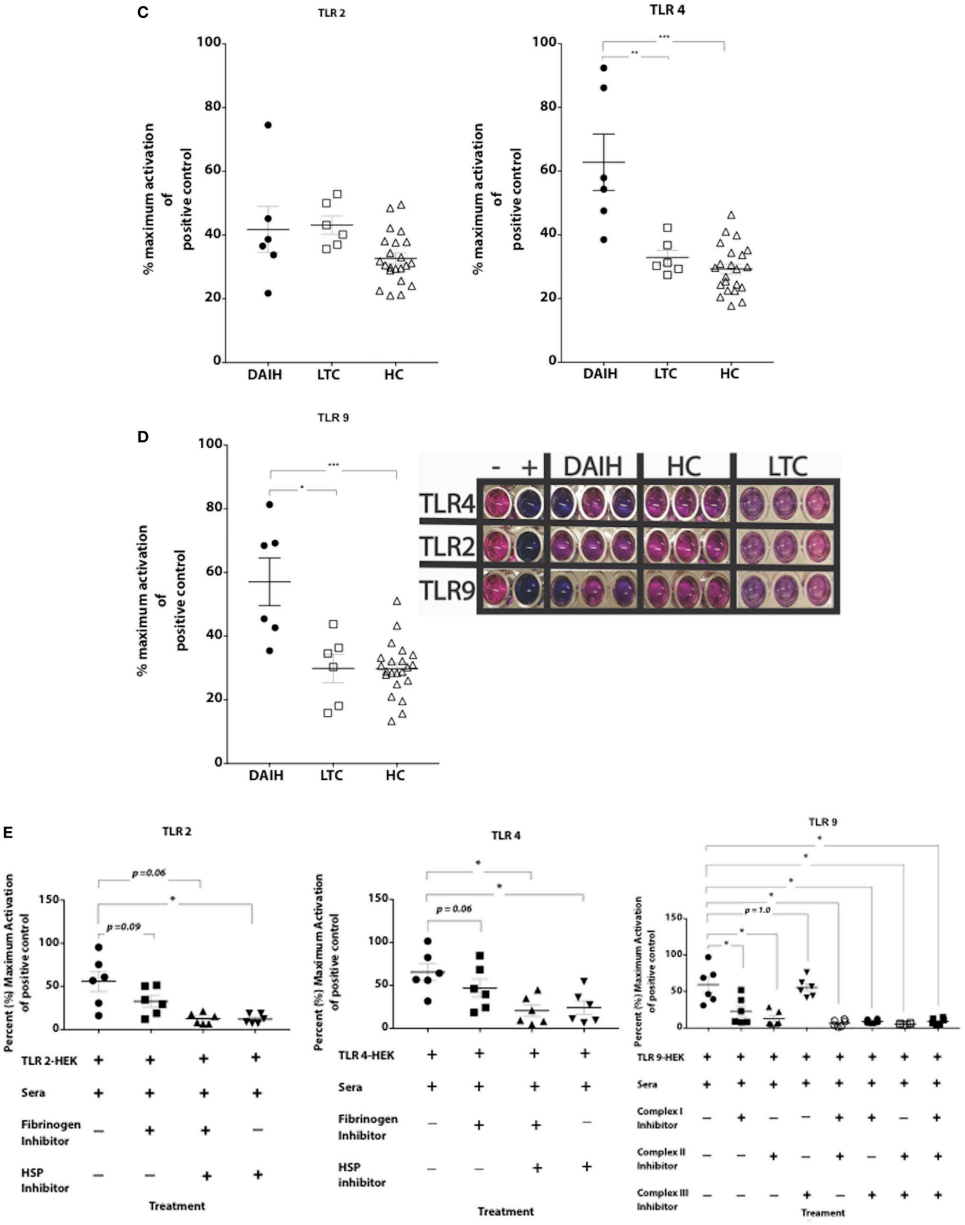
Damage-associated molecular patterns (DAMPs) providing inflammatory signal to the receptors toll-like receptor (TLR) 2/4 and 9, are significantly increased in sera of patients with *de novo* autoimmune hepatitis (DAIH). Sera from liver transplanted patients: with DAIH (*n* = 10), without DAIH (LTC) (*n* = 19); healthy, non-transplanted children (HC) (*n* = 29), and non-transplanted children with autoimmune hepatitis (AIH) (*n* = 9), was subjected to (i) qPCR; (ii) ELISA. HEK cells that stably co-express a human TLR2, TLR4, or TLR9 gene and an NF-κB-inducible secreted embryonic alkaline phosphatase (SEAP) reporter gene were used to determine activation of TLRs from patient sera. **(A,B)** significant abundance of DAMPs that activate TLRs 2/4 and 9 and levels of cytosolic proteins associated with TLR 2/4 activation in sera of patients with DAIH compared to sera of LTC subjects (qPCR: *p* < 0.001 for ACTB; *p* < 0.001 for MT-ATP/complex I; *p* < 0.001 for Fibrinogen; *p* < 0.001 for HMGB1, *p* < 0.001 for HSP70; and *p* < 0.001 for HSP90, *p* = 0.05 for HSP60) (ELISA: *p* = 0.003 for Fibrinogen; *p* = 0.37 for HMGB1). Significant abundance of DAMPs that activate TLRs 2/4 and 9 and levels of mitochondrial, cytosolic, and functional proteins in sera of patients with DAIH compared to sera of HC subjects (qPCR: *p* < 0.001 for Fibrinogen, HMGB1, HSP70, HSP90, ACTB, and MT-ATP6, respectively, and *p* = 0.64 for HSP60). Significant abundance of DAMPs that activate TLRs 2/4 and 9 and levels of functional proteins in sera of patients with AIH compared to sera of HC subjects. [qPCR: *p* < 0.001 for ACTB (nuclear DNA); *p* = 0.002 for MT-ATP6 (mitochondrial DNA); *p* = 0.013 for Fibrinogen; *p* = 0.008, *p* = 0.005, and *p* = 0.008 for HSPs 60, 70 and 90, respectively] (ELISA: *p* = 0.043 for HMGB1; *p* = 0.11 for fibrinogen). ***p < 0.001, **p < 0.01, *p < 0.05. **(C,D)** Activation of TLRs 2, 4, and 9 reporter cell lines by sera of patients with DAIH. DAIH vs. LTC: (*p* = 0.47; TLR2) (*p* = 0.008; TLR4) (*p* = 0.02; TLR9). DAIH vs. HC: (*p* = 0.17; TLR2) (*p* < 0.001; TLR4) (*p* = 0.001; TLR9). Minus sign: negative control, plus sign: positive control. Representative plate for three patients from each subject group and summary data. **(E)** Protein inhibition targeting heat shock protein, fibrinogen, and complexes I–III significantly reduced activation of TLR2/4 and TLR9 reporter cell lines, respectively [for TLR 9 reporter cell line inhibition: *p* = 0.03 for Rotenone (Complex 1), *p* = 0.03 for Thenoyltrifluoroacetone, TTFA (Complex II), *p* = 1.0 for Antimycin A (Complex III), *p* = 0.03 for Complexes I and II, *p* = 0.03 for Complexes I and III, *p* = 0.03 for Complexes II and III, *p* = 0.03 for Complexes I, II, and III; for TLR 4 reporter cell line inhibition: *p* = 0.06 for fibrinogen inhibitor, *p* = 0.03 for the HSP90 inhibitor, 17-*N*-Allylamino-17-demethoxygeldanamycin, 17AAG, and *p* = 0.03 for both HSP90 and fibrinogen inhibitors; for TLR 2 reporter cell line inhibition: *p* = 0.09 for Fibrinogen inhibitor, *p* = 0.03 for the HSP90 inhibitor, 17AAG, *p* = 0.06 for both HSP90 and fibrinogen inhibitors]. (HSP—heat shock protein; positive controls: FSL-1—synthetic diacylated lipoprotein, LPS—lipopolysaccharide, ODN 2006—CpG oligonucleotide). Summary data.

To further understand the mechanism underlying inflammasome activation in DAIH, we looked for the presence of DAMPs in sera and found sera of patients with DAIH had a significant abundance of DAMPs known to activate TLRs 2/4 and 9 (3) (Figures 2A,B) compared to sera of LTC subjects, and compared to sera of healthy, non-transplanted children (HC). Similarly, in non-transplanted children with AIH, DAMPs that activate TLR’s, 2, 4, and 9 were significantly increased in sera compared to sera of healthy children with no underlying immune-mediated disorders (HC) (Figures 2A,B). Using TLR reporter cell lines, we confirmed significantly greater activation of TLR 4 and TLR 9 by sera of patients with DAIH (compared to sera of LTC and HC subjects) (Figures 2C,D). Furthermore, protein inhibition, targeting specific DAMPs—(Fibrinogen, HSPs, and Complexes I–III), effectively reduced TLRs 2/4 and 9 activation by sera of patients with DAIH (Figure 2E) (Figure S1C in Supplementary Material). Together these results are consistent with our interpretation that TLRs 2/4 and 9 mediates activation of the inflammasome in CD14^++^ monocytes of patients with DAIH.

Similarly, sera from children with AIH also activated TLR 2, 4, and 9 reporter cell lines (Figure S1D in Supplementary Material) further confirming that activation of the inflammasome takes place in CD14^++^ monocytes from patients with AIH, and is mediated *via* TLRs 2, 4, and 9. The above observation supports the notion that post-transplant DAIH and AIH are similar diseases and may share a common disease pathogenesis. To address concerns that release of DAMPs could be related to elevated serum alanine aminotransferase (ALT) levels from hepatocyte lysis, median ALT values in subject groups were compared and no significant difference in ALT values was observed between DAIH and LTC subjects (Table S2 in Supplementary Material). Moreover, there was no significant correlation between DAMPs release and ALT for DAIH subjects (Table S3 in Supplementary Material). We also found no significant positive correlation between DAMPs and ALT in subjects with AIH; however, there was a significant negative correlation between mitochondrial DNA and ALT in subjects with AIH (Figure S1E in Supplementary Material). Taken together, the above supports our interpretation that release of DAMPs is related to disease and not hepatocyte lysis.

### Stimulation of TLRs 2 and 4 in CD14^++^ Monocytes Promotes Th1-Like Treg Phenotype

**Figure 3:**
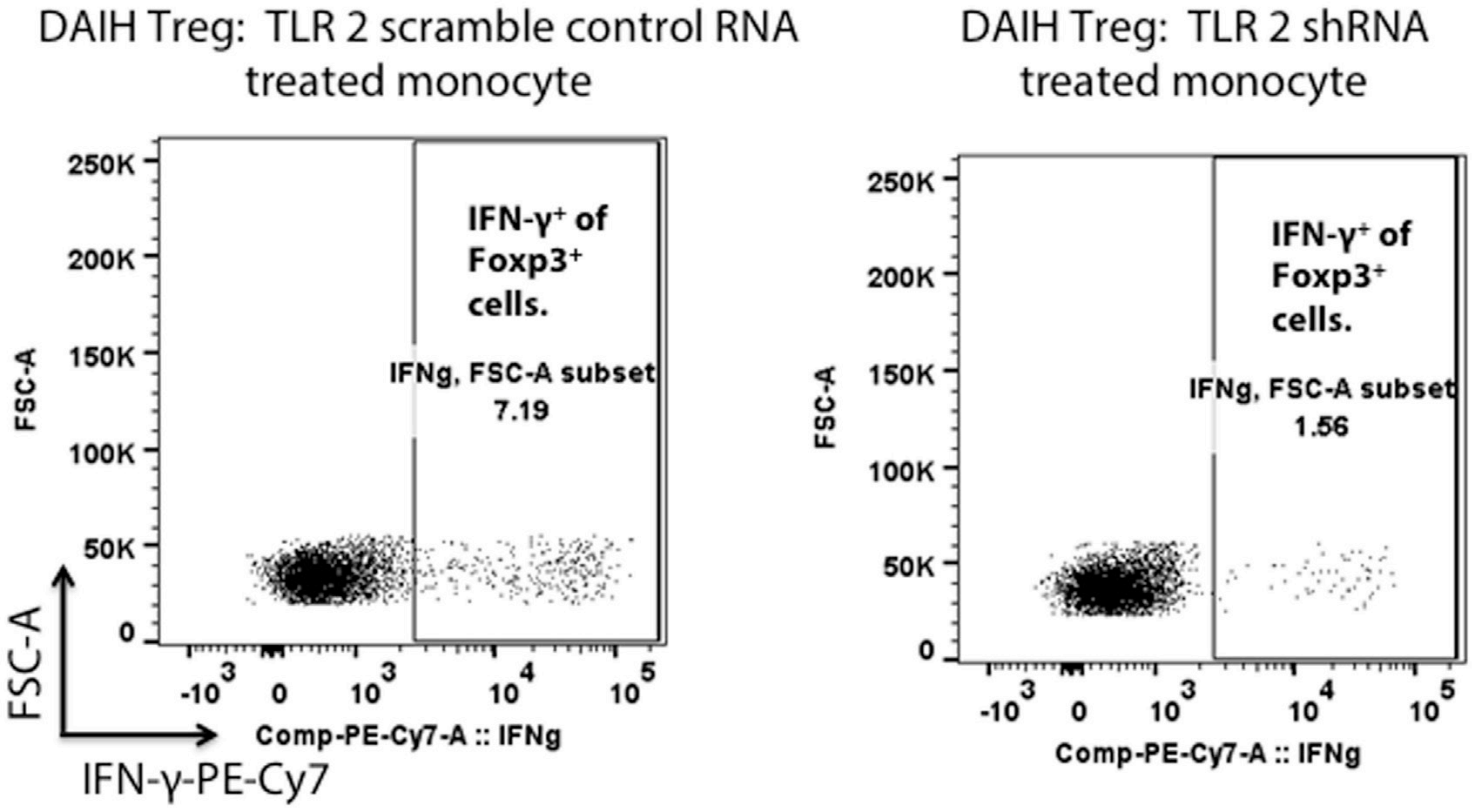
Silencing of toll-like receptors (TLRs) 2 and 4 in CD14^++^ monocytes of patients with *de novo* autoimmune hepatitis (DAIH) prevents IL-12-mediated regulatory T cell (Treg) differentiation to Th1-like Tregs. CD14^++^ monocytes from patients with DAIH (*n* = 4) were subjected to shRNA inhibition of TLR 2/4 and 9 and then co-cultured with sorted CD4^+^CD25^hi^CD127^neg^ FOXP3^+^ Tregs from patients with DAIH and HC subjects, in the presence of plate bound anti-CD3 for 5 days and IFN-γ production from FOXP3^+^ Tregs was assessed using flow cytometry. Representative flow cytometry plot showing reduced IFN-γ production from FOXP3^+^ Tregs of DAIH patients following co-culture of their sorted Tregs with their silenced TLR 2 monocytes and scramble control monocytes.

To confirm the role of the innate immune system in driving Treg differentiation, shRNA inhibition of TLRs 2/4 and 9 in CD14^++^ monocytes was done followed by co-culture of the monocytes with FOXP3^+^ Tregs as previously described (1). We confirmed shRNA inhibition of TLRs 2/4 and 9 in CD14^++^ monocytes (Figures S2A–D in Supplementary Material). Silencing of TLRs 2 and 4 in CD14^++^ monocytes resulted in significantly less IFN-γ production by FOXP3^+^ Tregs (Figure 3; Figure S3C in Supplementary Material), however, silencing of TLR 9 did not significantly reduce IFN-γ production by FOXP3^+^ Tregs (Figure S3D in Supplementary Material) (gating strategy Figure S3A in Supplementary Material). The above confirms that TLR 2 and TLR 4 mediated activation of CD14^++^ monocytes drives Treg differentiation to pro-inflammatory, Th1-like effector Tregs in DAIH.

### Increased Expression of Inflammasome-Associated Components in DAIH Liver

**Figure 4:**
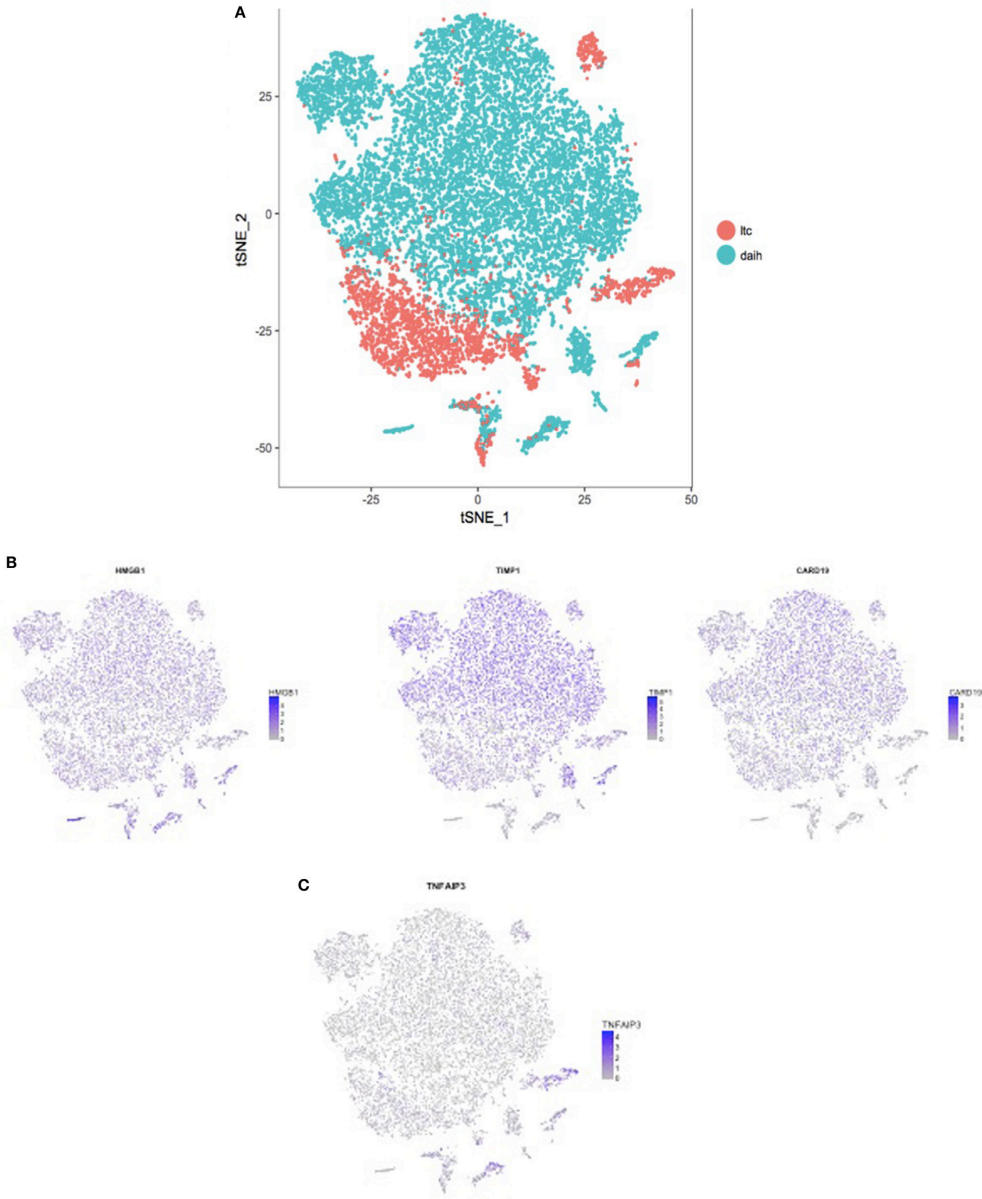

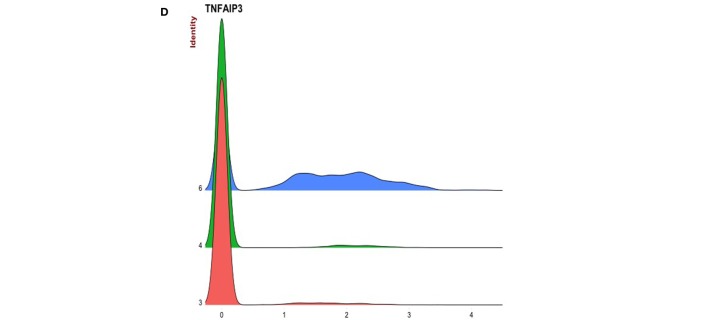
CD14^++^ monocytes from peripheral blood mononuclear cells (PBMCs) and CD68^+^ macrophages from the liver of patients with *de novo* autoimmune hepatitis (DAIH) display increased expression of inflammasome-associated components and absence of a negative regulator of infammasome activation. PBMCs from blood and intrahepatic lymphocytes from the liver were obtained from liver transplanted patients with DAIH (*n* = 5), and liver transplanted patients with normal allograft function who do not have DAIH (LTC) (*n* = 4), stained for CD3CD14 (PBMC) and CD45CD68 (intrahepatic lymphocytes), and the monocyte (CD3^−^CD14^++^ PBMC) and macrophage (CD45^+^CD68^+^ liver) population were then sorted on a FACS Aria and subjected to library preparation for single-cell sequencing. After log normalizing and scaling the data, variable genes were detected using Seurat (10). These highly variable genes were used to generate principal components, and cluster detection was done using Seurat. The clusters were visualized using the tSNE rendering in Seurat. **(A)** CD14^++^ monocytes and CD68^+^ macrophages from subjects with *de novo* autoimmune hepatitis cluster together and away from monocytes and macrophages of LTC subjects. **(B)** After cluster detection, marker genes for each cluster were detected such that the gene(s) is expressed in at least 25% of the cells in the given cluster, and it is overexpressed by at least 25% than all of the rest of the cells. The expression values for these genes were then plotted in each cell, and rendered according to the tSNE to give the feature plots. Feature plots showing significant overexpression of *HMGB1* (*p* = 2.62 × 10^−69^, fold > 3), *CARD19* (*p* = 2.13 × 10^−59^, fold > 2), and *TIMP1* (*p* = 1.03 × 10^−79^, fold > 3) in clusters 3 + 4 compared to cluster 6 i.e., significant overexpression in monocytes/macrophages of subjects with DAIH compared to monocytes/macrophages of LTC subjects. **(C)** Feature plot showing significant overexpression of tumor necrosis factor α-induced protein 3 (*TNFAIP3*) (*p* = 3.44 × 10^−233^, fold > 2) in cluster 6 compared to clusters 3 + 4 i.e., significant overexpression in monocytes/macrophages of LTC subjects compared to monocytes/macrophages of subjects with DAIH. **(D)** Joy plot confirms the near absence of *TNFAIP3* from monocytes/macrophages of patients with DAIH and its high expression in monocytes/macrophages of LTC subjects.

To test the hypothesis that inflammasome activation is also present in the liver of subjects with DAIH, CD68^+^ macrophages and CD14^++^ monocytes were FACS sorted from liver and blood, respectively and subjected to single-cell sequencing. Absolute cell numbers varied between 126 and 9,494 cells (Table S1 in Supplementary Material). We observed that both CD14^++^ monocytes (blood) and CD68^+^ macrophages (liver) obtained from patients with DAIH clustered together and away from monocytes and macrophages obtained from liver transplanted subjects with normal allograft function (LTC) suggesting that CD14^++^ monocytes and CD68^+^ macrophages obtained from subjects with DAIH are indeed very similar in terms of their transcriptional profile (Figure 4A). Upon closer examination of individual clusters of cells, the cells within clusters 3 and 4 contained CD14^++^ monocytes and CD68^+^ macrophages from subjects with DAIH while cells within cluster 6 contained CD14^++^ monocytes and CD68^+^ macrophages from LTC subjects (Figures S4A,B in Supplementary Material). We then proceeded to compare clusters 3 + 4 vs. cluster 6. There were 135 genes significantly overexpressed in the clusters 3 + 4 vs. cluster 6 comparison and 258 genes significantly overexpressed in the cluster 6 vs. clusters 3 + 4 comparison (Figure S4C in Supplementary Material). Some of the significantly expressed genes in clusters 3 + 4 cells (i.e., DAIH monocytes/macrophages) compared to cluster 6 cells (i.e., LTC monocytes/macrophages) include *HMGB1*, which provides inflammatory signal 1 to TLR 4 in the two-signal activation of the inflammasome machinery (3), *CARD19*, the caspase recruitment domain (CARD) is present in death-domain superfamily proteins and involved in inflammation and apoptosis (7). Importantly, the inflammasome complex includes a CARD containing protein (8), and *TIMP1* (Figure 4B). In the cluster 6 vs. clusters 3 + 4 comparison, tumor necrosis factor α-induced protein 3 (*TNFAIP3*), a negative regulator of the NLRP3 inflammasome (9), was significantly expressed in cluster 6 (i.e., LTC monocytes/macrophages) and almost absent in clusters 3 + 4 (i.e., DAIH monocytes/macrophages) (Figures 4C,D). We believe that these observations support a role for inflammasome activation in disease pathogenesis of DAIH. To support our interpretation of common features in underlying pathogenesis of both DAIH and AIH, we subjected CD14^++^ monocytes of non-transplanted subjects with AIH to single-cell RNA-sequencing and observed 1 cluster of AIH cells that appeared associated with the DAIH cluster of cells; additionally, some AIH cells intermingled with DAIH cells, however, this percentage was low (Figure S4D in Supplementary Material). Upon closer examination of individual cluster of cells, cluster 3 has AIH cells and cluster 11 has cells from liver transplanted subjects with normal allograft function who do not have DAIH (LTC) (Figure S4E in Supplementary Material); specifically, cluster 3 has 90% proportion of AIH cells, 10% proportion of DAIH cells while cluster 11 has 90% proportion of LTC cells (Figure S4F in Supplementary Material). We therefore proceeded to compare cluster 3 vs. cluster11. *TIMP1* and the heat shock family of proteins were significantly overexpressed in the cluster 3 cells vs. cluster 11 comparison: *TIMP1*: average logFC 2.33 *p* = 9.0 × 10^−223^; *HSP90*: average logFC 0.4 *p* = 1.15 × 10^−95^ (Figure S4G in Supplementary Material). We believe the above supports a role for inflammasome activation in disease pathogenesis of AIH.

The most important advantage of single-cell RNA-sequencing is the ability to study transcriptomes of populations of cells. Unlike FACS that solely depends on surface markers, single-cell RNA-seq allows us to study the heterogeneity in the population of cells that might have identical cell surface markers. As our samples are from different patients and thus have an *n* that is greater than 1, they are not technical replicates Therefore, even small populations of cells that show specific transcription profiles can still provide statistically robust and biologically meaningful results.

### Hepatocyte Apoptosis Is Induced by CD14^++^ Monocytes

**Figure 5:**
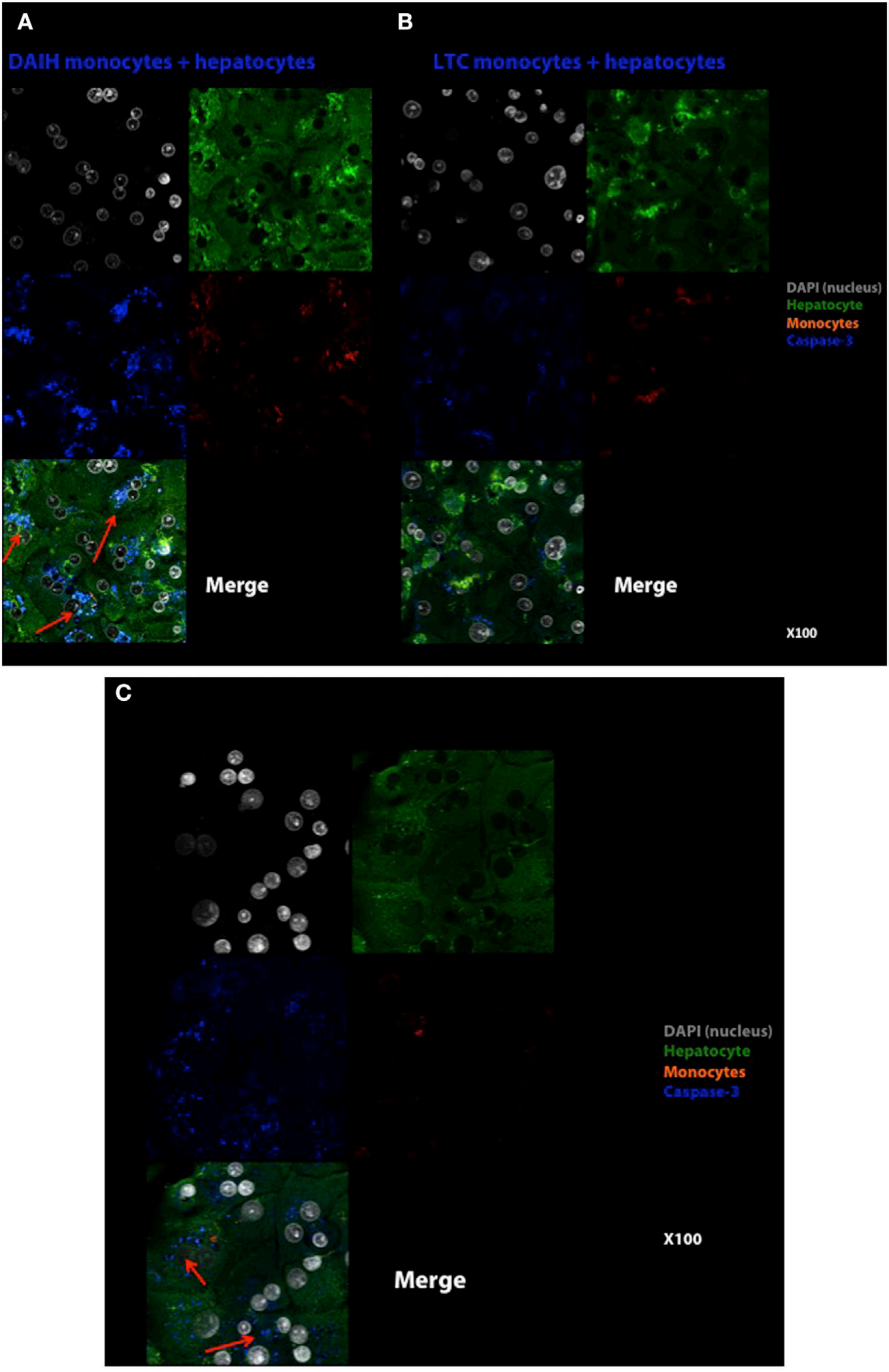
CD14^++^ monocytes from patients with *de novo* autoimmune hepatitis (DAIH) induce hepatocyte perturbation as evidenced by caspase-3 cleavage of hepatocytes. CD14^++^ monocytes from liver transplanted patients with DAIH (*n* = 2), liver transplanted patients with normal allograft function who do not have DAIH (LTC) (*n* = 2), and non-transplanted children with autoimmune hepatitis (AIH) (*n* = 2) were isolated by negative selection and co-cultured with normal hepatocytes over 96 h, and anti-active caspase-3 production by hepatocytes were assessed by confocal microscopy. **(A)** Hepatocytes cultured with DAIH monocytes for 96 h. DAIH monocytes induce hepatocyte apoptosis (arrows). Top left: DAPI (nucleus)—gray, top right: hepatocytes—green, middle left: caspase-3—blue, middle right: monocytes—orange, bottom left: merge. 100× magnification. **(B)** Hepatocytes cultured with LTC monocytes for 96 h. Top left: DAPI (nucleus)—gray, top right: hepatocytes—green, middle left: caspase-3—blue, middle right: monocytes—orange, bottom left: merge. 100× magnification. **(C)** Hepatocytes cultured with AIH monocytes for 96 h. AIH monocytes induce hepatocyte apoptosis (arrows). Top left: DAPI (nucleus)—gray, top right: hepatocytes—green, middle left: caspase-3-blue, middle right: monocytes—orange, bottom left: merge. 100× magnification.

In addition to driving Treg differentiation, we sought to determine if the histological inflammation observed in DAIH is induced by CD14^++^ monocytes. To this end, CD14^++^ monocytes from PBMCs were co-cultured over 96 h with normal human hepatocytes. The readout was anti-caspase-3 from hepatocytes and alanine aminotransferase secretion in culture supernatant. Hepatocytes cultured alone did not induce hepatocyte apoptosis at 24 h (Figure S5A in Supplementary Material). After 96 h of hepatocyte co-culture with CD14^++^ monocytes obtained from subjects with DAIH, we observed hepatocyte apoptosis (Figure 5A), more so than following co-culture with CD14^++^ monocytes obtained from LTC subjects (Figure 5B). This induction of hepatocyte apoptosis at 96 h by CD14^++^ monocytes from subjects with DAIH was accompanied by elevation of alanine aminotransferase in the culture supernatant (Figure S5C in Supplementary Material). CD14^++^ monocytes from LTC subjects and healthy children did not induce an elevation in alanine aminotransferase in culture supernatant following 72 and 96 h of co-culture with hepatocytes (Figure S5C in Supplementary Material). This result suggests CD14^++^ monocytes from subjects with DAIH may cause select forms of hepatocyte injury. Perhaps not surprisingly, CD14^++^ monocytes obtained from non-transplanted subjects with AIH also induced hepatocyte apoptosis following a 96-h co-culture period (Figure 5C), however, this was not accompanied by elevations in alanine aminotransferase in culture supernatant (Figure S5C in Supplementary Material). As expected, CD14^++^ monocytes from healthy children did not induce hepatocyte apoptosis (Figure S5B in Supplementary Material).

**Figure 6:**
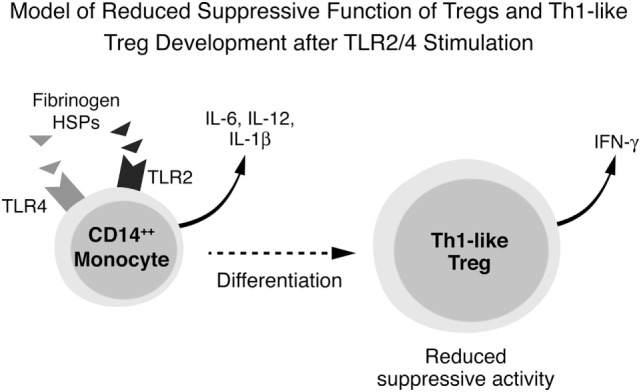
Conceptual framework for immune pathogenesis of *de novo* autoimmune hepatitis (DAIH). Damage-associated molecular patterns (DAMPs) in sera of patients with DAIH activate toll-like receptors (TLRs) 2, 4, and 9 in CD14^++^ monocytes, leading to activation of the inflammasome, caspase-1 cleavage, pro-IL-1β processing to IL-1β and IL-1β release. Activation of monocytes is also accompanied by production of other inflammatory cytokines including IL-12. In the presence of regulatory T cells (Tregs), IL-12 drives Tregs to secrete IFN-γ and thus become Th1-like Tregs that lack suppressive function (1, 11, 12). Inhibition of TLR 2 and TLR 4 (but not TLR 9) prevents secretion of IL-12 and thus prevents differentiation of Tregs to Th1-like Tregs.

These results lead us to propose the following as the underlying immune pathogenesis in DAIH: DAMPs activate TLR2, TLR4 and TLR9 in CD14^++^ monocytes promoting inflammasome activation with inflammatory cytokine secretion, including IL-12, from CD14^++^ monocytes. IL-12 drives Treg differentiation with a resultant dramatic increase in IFN-γ secretion from Tregs. These IFN-γ secreting Tregs (also called Th1-like Tregs) have previously been shown to lack the ability to suppress T effector cell proliferation (1, 11, 12). Inhibition of TLR2 and TLR4 (but not TLR 9) activation abrogates secretion of IL-12 thus preventing differentiation of Tregs to IFN-γ secreting Tregs (Figure 6).

## Discussion

The main finding in our study is that TLRs 2- and 4-mediated inflammasome activation in CD14^++^ monocytes is key to maintaining dysfunctional Tregs in liver transplanted patients with DAIH. Our study is the first to relate inflammasome activation in CD14^++^ monocytes to Treg dysfunction in DAIH. The inflammasome complex is a critical mediator in various autoimmune diseases; Moreover, it is thought to be a pivotal pathway connecting tissue injury with inflammation and autoimmunity (13). This is elegantly demonstrated in mice with *Abca1/g1* deficiency in dendritic cells (DCs) that have a lupus-like phenotype. Their CD11b^+^ DC population displays inflammasome activation and enhanced secretion of the inflammatory cytokines, IL-1β and IL-18, leading to skewing of T cells toward a Th1 phenotype. Deficiency of the NOD-like receptor family *pyrin domain* containing 3 (*NLRP3*) partly reversed some of the lupus-like features, as well as the increase in IL-1β and Th1 expansion indicating that this pathway contributes to aspects of the autoimmunity phenotype in DC-*Abca1/g1* deficiency(14).

In agreement with Westerterp et al. (14), our current work demonstrates that CD14^++^ monocytes of patients with DAIH display inflammasome activation mediated *via* TLR2 and TLR4, with enhanced IL-12 secretion that drives IFN-γ secretion from Tregs and Treg dysfunction. TLR2 and TLR4 silencing in CD14^++^ monocytes of these patients prevents Treg differentiation to Th1-like Tregs and thus IFN-γ secretion by Tregs of these patients (Figure 3), suggesting that autoimmunity is promoted by CD14^++^ monocytes predominantly through activation of inflammatory signaling pathways.

Further support for involvement of inflammasome activation in the pathogenesis of DAIH is illustrated by sera from patients with DAIH activating TLR2, TLR4, and TLR9 reporter cell lines (Figures 2C,D), and protein inhibition targeting specific DAMPs effectively reducing this activation of TLR reporter cell lines (Figure 2E), indicating an essential contribution of the inflammasome to key aspects of the autoimmune phenotype in DAIH. Moreover, we observed significantly increased expression of HMGB1 by CD68^+^ macrophages of subjects with DAIH (Figure 4B). TLR 4 appears to be the dominant receptor for HMGB1-mediated activation of macrophages (15). Direct involvement of inflammasomes in autoimmunity has been described in vitiligo (16, 17), systemic lupus erythematosus (SLE) (18, 19), and experimental autoimmune encephalomyelitis (20, 21). In addition, IL-18, the other effector cytokine of inflammasome-mediated caspase-1 activation has been implicated in the dysfunction of endothelial progenitor cells in SLE, impairing vascular repair (22). IL-18 also contributes to secondary progressive multiple sclerosis and rheumatoid arthritis joint inflammation, stimulating leukocyte chemotaxis, angiogenesis and cartilage destruction (23, 24).

Both priming and activation of inflammasomes require tight regulation to prevent an excessive inflammatory response. Inflammasome pathways are negatively regulated at multiple levels. Recently, A20/TNFAIP3 was found to be a negative regulator of the NLRP3 inflammasome, and specific deletion of A20 in myeloid cells resulted in spontaneous arthritis in mice that resembled human rheumatoid arthritis (9). We have demonstrated significantly increased expression of *TNFAIP3* in monocytes/macrophages of liver transplanted subjects without DAIH who have normal allograft function (LTC) (Figures 4C,D), and whose monocytes do not undergo inflammasome activation (Figures 1C–E) further supporting direct involvement of the inflammasome in pathogenesis of DAIH.

We questioned if the inflammation observed histologically in patients with DAIH is induced in part by their CD14^++^ monocytes. To overcome the limitation of the lack of an animal model of DAIH, we co-cultured CD14^++^ monocytes of patients with DAIH with normal human hepatocytes to observe if these monocytes could change hepatocyte survival. Following 96 h of co-culture, CD14^++^ monocytes from patients with DAIH induced apoptosis of normal hepatocytes (Figure 5A). The induction of apoptosis was accompanied by an increase in alanine aminotransferase levels in the culture supernatant (Figure S5C in Supplementary Material). Interestingly, even though CD14^++^ monocytes from non-transplanted subjects with AIH also induced hepatocyte apoptosis at 96 h (Figure 5C), it was not accompanied by alanine aminotransferase elevation (Figure S5C in Supplementary Material). This could be due to the fact that in AIH, a more prolonged co-culture period may be necessary to demonstrate accompanying liver enzyme elevation in the culture supernatant. Alternatively, it is also possible that their current treatment may result in normalization of liver biochemistry but not complete normalization of histological activity. Hepatocyte injury as a consequence of CD14^++^ monocyte actions has been reported by Nishio et al. (25) who observed that CD14^++^ monocyte derived galectin-9 increasing the toxicity of natural killer cells in chronic hepatitis C, with resultant liver injury and persistent infection.

There are several limitations to our study; first, we performed the TLR silencing experiments using CD14^++^ monocytes from blood and not CD68^+^ macrophages from the liver However, there is an enhanced ability of monocytes to enter the liver from blood through hepatic endothelium as reported by Liaskou et al. who show monocyte transendothelial migration across inflamed hepatic sinusoidal endothelium *in vitro* (26). In addition, monocytes circulate in blood for several days before migrating into tissues where they can differentiate into tissue macrophages (27). Co-expression of CD14, CD16, and CD68 on a population of large cells with the morphology of macrophages is present throughout the liver sinusoids confirming that distinct monocyte-derived and sessile macrophage populations coexist in the liver (26). Moreover, we have previously demonstrated co-expression of CD14^+^CD68^+^ cells in the liver of patients with DAIH confirming co-existence of monocyte-derived and tissue macrophages in the liver as well as suggesting that these monocytes are recruited from the blood (1). Second, our findings could be related to having a transplanted allograft and not necessarily the underlying disease. However, we have shown the absence of TLR 2 and 4-mediated inflammasome activation in CD14^++^ monocytes of similarly liver transplanted subjects who do not have DAIH. Furthermore, CD14^++^ monocytes of non-transplanted subjects with AIH also undergo TLRs 2, 4, and 9-mediated inflammasome activation supporting our contention of involvement of inflammasome activation in the pathogenesis of DAIH. Moreover, the biochemical (presence of autoantibodies and immunoglobulin G), histological features (peri-portal lympho-plasmacytic inflammation with interface hepatitis), and treatment of AIH and DAIH are the same. Third, the lack of a suitable animal model of DAIH has precluded us from demonstrating our findings *in vivo*. It is possible that co-culture of DAIH CD14^++^ monocytes + Tregs with hepatocytes for a prolonged time period may result in even more hepatocyte perturbation with aminotransferase elevation than we have demonstrated, perhaps similar to that seen in the serum of untreated subjects with DAIH or that co-culture of these monocytes + Tregs with cholangiocytes may result in bile duct injury similar to that reported in subjects with DAIH (4, 28). If this is clearly demonstrated, then addressing prevention of inflammasome activation either by inhibition of TLR 2 and TLR 4 or protein inhibition targeting specific DAMPs would need to be investigated in humans.

## Conclusion

Priming of inflammasomes is increased in both DAIH and AIH due to innate immune stimuli released from damaged cells. Understanding the regulatory network of inflammasome activation in these two diseases will shed light on the development of new target therapy. In addition, large population genetic association studies looking at single-nucleotide polymorphisms in inflammasome components in these two diseases would be helpful to validate our current data.

## Ethics Statement

This study was carried out in accordance with the recommendations of [Yale Institutional Review Board], [Human Investigation Committee]. The protocol was approved by the [Human Investigation Committee]. All subjects gave written informed consent in accordance with the Declaration of Helsinki.

## Author Contributions

AA: concept design, performed experiments, data analysis, and writing first draft of manuscript; JY and AL: performed experiments, data analysis, and manuscript review; YA, MM, and SL: subject recruitment, sample collection, review of inclusion/exclusion criteria, and manuscript review; YD and GG: statistics, data analysis, and manuscript review; SM and JK: single-cell sequencing data analysis, manuscript writing, and manuscript review; GW: single cell sequencing, single cell sequencing data analysis, manuscript writing, and manuscript review; UE: concept design, data analysis, revising first and subsequent drafts of manuscript, subject recruitment, sample collection, and review of inclusion/exclusion criteria.

## Conflict of Interest Statement

The authors declare that the research was conducted in the absence of any commercial or financial relationships that could be construed as a potential conflict of interest.
