# Supplementary material for: Inflammasome Priming Mediated via Toll-Like Receptors 2 and 4, Induces Th1-Like Regulatory T Cells in De Novo Autoimmune Hepatitis
Source: Front Immunol. 2018 Jul 19;9:1612. doi: 10.3389/fimmu.2018.01612 (PMC6060440; doi:10.3389/fimmu.2018.01612)
Supplement: Supplementary file 1 [file Data_Sheet_1.PDF]

Supplementary Material

Inflammasome Priming Mediated Via Toll-Like Receptors 2 And 4, Induces Th1-Like Tregs In

De Novo Autoimmune Hepatitis.

Adam Arterbery, PhD[1], Jie Yao, PhD[1], Andrew Ling, BSc[1], Yaron Avitzur, MD[2], Mercedes Martinez, MD[3], Steven Lobritto, MD[3], Yanhong Deng, MPH[4], Gan Geliang, PhD[4], Sameet Mehta, PhD[5], Guilin Wang, PhD[5], James Knight, PhD[5], *Udeme D. Ekong, MD, MPH[1].

*** Correspondence:** Udeme D. Ekong MD MPH

Section of Pediatric Gastroenterology and Hepatology

333 Cedar Street, LMP 4093, PO Box 208064

New Haven, CT 06520

Tel: 203 785 4649

Fax: 203 785 3365

Email: udeme.ekong@yale.edu

1 Supplementary Data

Antibodies and reagents

For cell surface staining: anti-CD4 (clone RPA-T4; BD Pharmingen); CD25 (clone M-A251; BD Pharmingen); CD127 (clone eBioRDR5; eBioscience); anti-CD3 (clone UCHT1; BD Biosciences); anti-CD14 (clone MfP9; BD Biosciences).

For intracellular staining: anti–IFN-γ (clone 4S.B3; BioLegend); Foxp3 (clone PCH101; eBioscience); anti–IL-12 (clone [11C] 5; BD Biosciences); anti–IL-1β (clone AS10; BD Biosciences); Live Dead – Near IR fluorescent Reactive Dye (L10119, Life Technologies).
For western blotting: NLRP3 (Cryopyrin, sc-66846, Santa Cruz Bio); ASC (sc-30153, Santa Cruz Bio); CASP-1 (sc-515, Santa Cruz Bio); IL-1β (sc-7884, Santa Cruz Bio); GAPDH (sc-25778, Santa Cruz Bio). Reagents: Digital anti-rabbit-HRP (Kindle Bio); Omniblock (American Bio); Super Digital ECL (Kindle Bio); PhosSTOP phosphatase inhibitor cocktail (Roche); Pre cast 4-12% Bis-Tris Gels (Gen10 Bio); MPER (Mammalian Protein Extraction Reagent, 78501, Thermo Scientific); 4x Lamaelli (1610747, Bio-Rad); NuPAGE™ MES SDS Running Buffer (20X, NP0002,

Invitrogen); NuPAGE™ Transfer Buffer (20X, NP0006, Invitrogen); 2-mercaptoethanol (M6250-10ML, Sigma Aldrich); Immun-Blot PVDF Membrane/Filter Paper Sandwiches (**1620238,** Bio-Rad).

For measurement of DAMPs in sera - qPCR: *Pro-IL-1β* (Hs00174097_m1, Applied Biosystems); *HMGB1* (Hs01037385_s1, Applied Biosystems); *ACTB* (Hs01060665_g1, Applied Biosystems); *MT-ATP6* (Hs02596862_g1, Applied Biosystems); *Fibrinogen* (Hs00189514_m1, Applied Biosystems); *HSP70* (HSPA1A, Hs00359163_s1, Applied Biosystems); *HSP60* (HSPD1, Hs01036753_g1, Applied Biosystems); *HSP90* (HSP90AA, Hs00743767_sH, Applied Biosystems); *β2M* (Hs00984230_m1, Applied Biosystems); 18s (Hs99999901_s1, Applied Biosystems).

For genes - qRT-PCR: *TLR2* (Hs02621280_s1, Applied Biosystems); *TLR4* (Hs00152939_m1, Applied Biosystems); *TLR9* (Hs00370913_s1, Applied Biosystems).

Reagents: SsoAdvanced Universal Probes Supermix (**1725281,** Bio-Rad); qScript cDNA Supermix (950048-100, QuantaBio); RNeasy Plus Micro Kit (74034, Qiagen); QIAamp DNA Blood Mini Kit (51104, Qiagen).

For Toll-like Receptor reporter cell lines (DAMPs inhibition): Rotenone (Complex I inhibitor, 150154, MP Bio); TTFA (Complex II inhibitor, 520442010, MP Bio); Antimycin A (Complex III inhibitor, ALX-380-075-M005, Enzo Life Sciences); HEK-Blue hTLR2 reporter cells (hkb-htlr2, Invivogen); HEK-Blue hTLR4 reporter cells (hkb-htlr4, Invivogen); HEK-Blue hTLR9 reporter cells (hkb-htlr9, Invivogen); QUANTI-Blue (rep-qb1, Invivogen); LPS (TLR4 positive control ligand, tlrl-peklps, Invivogen); FSL1 (TLR2 positive control ligand, tlrl-fsl, Invivogen); ODN 2006 (TLR9 positiive control ligand - ODN 7909, tlrl-2006, Invivogen); DMEM (+Glucose/+L-Glutamine, 11965-092, Gibco – Life Technologies); Heat Inactivated FBS (F4135-500ML, Sigma Aldrich); Penicillin-Streptomycin (15140122, Gibco – Life Technologies); Normocin (ant-nr-1, Invivogen); Blasticidin (ant-bl-05, Invivogen); Hygromycin (ant-hg-1, Invivogen); Zeocin (ant-zn-1, Invivogen).

TLR shRNA: TLR2 lentiviral shRNA Particles (TL320553V, Origene); TLR4 lentiviral shRNA Particles (TL320555V, Origene); TLR9 lentiviral shRNA Particles (TL301076V, Origene); TLR-shRNA lentiviral scrable control shRNA Particles (TL30021V, Origene); Polybrene (TR-1003-G, Sigma Aldrich); Recombinant human Interleukin-2 (hIL-2, 10799068001, Roche-Sigma Aldrich).

For cell selection/isolation: Human CD14 Positive Selection Kit (18058, STEMCELL Technologies); Human CD4+ T Cell Enrichment Kit (19052, STEMCELL Technologies).

For Luminex (ELISA): MILLIPLEX MAP Heat Shock Protein Magnetic Bead 5-Plex Kit (48-615MAG, EMD Millipore-Sigma); MILLIPLEX MAP Human Oxidative Phosphorylation (OXPHOS) Magnetic Bead Panel (H0XPSMAG-16K, EMD Millipore-Sigma); MILLIPLEX MAP Human Cardiovascular Disease (Acute Phase) Magnetic Bead Panel 3 (Fibrinogen 1 Plex, HCVD3MAG-67K; S100A12, EMD Millipore-Sigma).

For hepatocyte/CD14^{++} co-culture experiments: HMM maintenance media (Lonza, MD) containing matrigel® basement membrane (VWR, PA), insulin 100nM, antibiotic antimycotic 50μg/ml, dexamethasone 100nM (Sigma, MO).

Alfa Wassermann reagent kits for measurement of alanine aminotransferase (SA1046) (West Caldwell, NJ).

For immunofluorescence confocal microscopy: anti-active caspase-3 (ab32042), anti-CD14 (ab182030), anti-albumin (ab10241), Alexa Fluor 488-labeled IgG (ab150113), Alexa Fluor 555-labeled IgG (ab150070), Alexa Fluor 647-labeled IgG (ab150115), (Abcam, MA), collagen type 1 rat-tail (Millipore), trypsin-EDTA (GIBCO).

Materials and Methods

For IL-1β FACS: $CD14^{++}$ monocytes from liver transplanted patients with DAIH (n = 7) and without DAIH (LTC) (n = 10) and healthy, non-transplanted children (HC) (n = 14) were stimulated with LPS for 24-hours and subjected to: (i) staining with anti-CD3, anti-CD14, intracellular IL-1β. Cytokine secretion analyzed using flow cytometry.

For Western blot: $CD14^{++}$ monocytes from non-transplanted children with autoimmune hepatitis (AIH) (n = 8) were subjected to Western Blot for pro-caspase-1, caspase-1, pro-IL-1β, and IL-1β.

For TLR reporter cell line stimulation: HEK cells that stably co-express a human TLR2, TLR4, or TLR9 gene and an NF-κB–inducible secreted embryonic alkaline phosphatase (SEAP) reporter gene were used to determine activation of TLR by sera of non-transplanted children with AIH.

For TLR silencing experiments: TLR-specific shRNA lentiviral particles were administered to $CD14^{++}$ monocytes from liver transplanted patients with DAIH (n = 4), and subsequently stimulated with LPS for 24-hours, then (i) subjected to qPCR for TLR 2, TLR 4, TLR 9, pro-IL-1β, IL-6, and IL-12 gene expression; (ii) stained with anti-CD14, intracellular IL-1β, IL-6 and IL-12. Cytokine secretion analyzed using flow cytometry; (iii) subjected to Western Blot for pro-caspase-1, caspase-1, IL-1β.

For monocyte/Treg co-culture experiments: TLR 2, TLR 4, TLR 9-lentiviral silenced and scramble control treated $CD14^{++}$ monocytes from subjects with de novo autoimmune hepatitis (DAIH) (n = 4) were co-cultured with sorted Tregs from healthy, non-transplanted subjects (HC) (n = 4) or subjects with DAIH (n = 4) in the presence of plate bound anti-CD3 for 5-days and IFN-γ production from $FOXP3^{+}$Tregs was assessed using flow cytometry.

For monocyte/hepatocyte co-culture experiments: $CD14^{++}$ monocytes from healthy, non-transplanted subjects (n=2) were isolated by negative selection and co-cultured with normal hepatocytes over 24-hours, and anti-active caspase-3 production from hepatocytes was assessed by confocal microscopy.

Fig. S1. Gating strategy – IL-1β from $CD14^{++}$ monocytes.

A) Forward and side scatter identified monocyte population; single cells were next gated on using FSC-W vs. FSC-H and SSC-W vs. SSC-H, viable cells were gated on, and of these, $CD14^{++}$ and $CD3^{-}$cells were gated upon; of these SSC-A vs. IL-1β identified. Gate placement for cytokines determined by FMO.
B) Activation of the inflammasome observed in $CD14^{++}$ monocytes from non-transplanted children with AIH compared to monocytes from healthy children (HC) as evidenced by cleavage of caspase-1 ($p < 0.001$ for pro-caspase; $p < 0.001$ for caspase-1) and secretion of IL-1β ($p < 0.001$ for pro-IL-1β; $p < 0.001$ for IL-1β). Of note, significantly increased levels of pro-caspase, caspase-1, pro-IL-1β, and IL-1β observed even in the unstimulated monocytes compared to unstimulated monocytes from

HC subjects. ($p < 0.001$ for pro-caspase; $p < 0.001$ for caspase-1) ($p < 0.001$ for pro-IL-1β; $p < 0.001$ for IL-1β). Representative blot and summary graph for nine patients. ### Represents the AIH vs. HC comparison.

C) Protein inhibition targeting heat shock protein, fibrinogen and complex I-III. (HSP – heat shock protein; Positive controls: FSL-1 – synthetic diacylated lipoprotein, LPS – lipopolysaccharide, ODN 2006 - CpG oligonucleotide). Representative plate for three patients from each subject group.
D) Activation of TLR 2, 4 & 9 reporter cell lines by sera of patients with AIH. AIH vs. HC: ($p = 0.14$; TLR2) ($p < 0.001$; TLR4) ($p < 0.001$;TLR9). Representative plate and summary figure. Minus sign: Negative control, plus sign: positive control.
E) ALT negatively correlated with mitochondrial DNA in subjects with AIH (MT-ATP6: correlation coefficient = -0.73 p = 0.02).

Fig. S2. Silencing of TLR 2/4 & 9 in $CD14^{++}$ monocytes significantly reduces pro-inflammatory cytokine production as well as caspase-1 cleavage.

A) At 5-days, $CD14^{++}$ monocytes from patients with DAIH that had TLR-specific shRNA lentiviral administered particles expressed significantly less *TLR 2* ($p = 0.03$), *TLR 4* ($p = 0.04$), and *TLR 9* ($p = 0.02$). Closed bars: shRNA treated. Open bars: scramble control.

B) At 5-days, $CD14^{++}$ monocytes from patients with DAIH that had TLR-specific shRNA lentiviral administered particles expressed significantly less *pro-IL-1β* following TLR 2 and TLR 4 silencing but not following TLR 9 silencing. TLR 2 ($p = 0.03$), TLR 4 ($p = 0.04$), TLR 9 ($p = 0.3$). Closed bars: shRNA treated. Open bars: scramble control.

C) At 5-days, $CD14^{++}$ monocytes from patients with DAIH that had TLR-specific shRNA lentiviral administered particles produced significantly less pro-inflammatory cytokines compared to scramble control $CD14^{++}$ monocytes following silencing of TLR 2 and TLR 4 but not TLR9. [IL-1β ($p = 0.02$ for TLR 2, $p = 0.02$ for TLR 4, $p = 0.8$ for TLR 9); IL-6 ($p = 0.02$ for TLR 2, $p = 0.02$ for TLR 4, $p = 0.9$ for TLR 9); and IL-12 ($p = 0.02$ for TLR 2, $p = 0.02$ for TLR 4, $p = 0.2$ for TLR 9)].

D) TLR 2 and TLR 4-lentiviral silenced $CD14^{++}$ monocytes demonstrated absence of caspase-1 cleavage compared to scramble control treated TLR 2 and TLR 4-$CD14^{++}$ monocytes. TLR 9-lentiviral silenced $CD14^{++}$ monocytes continued to exhibit caspase-1 cleavage, similar to scramble control treated TLR 9-$CD14^{++}$ monocytes.
Representative blot for two patients.

Fig. S3. Silencing of TLR 2 & 4 in $CD14^{++}$ monocytes of patients with DAIH prevents IL-12 Mediated Treg differentiation to TH1-like Tregs.

$CD14^{++}$ monocytes from patients with DAIH (n = 4) were subjected to shRNA inhibition of TLR 2/4 & 9 and then co-cultured with sorted $CD4^{+}CD25^{hi}CD127^{neg}$ $FOXP3^{+}$ Tregs from patients with DAIH and HC subjects, in the presence of plate bound anti-CD3 for 5-days and IFN-γ production from $FOXP3^{+}$ Tregs was assessed using flow cytometry.

Gating strategy – $CD14^{++}$ monocyte and Treg co-culture experiments.

A-B) Forward and side scatter identified lymphocyte population; single cells were next gated on using FSC-W vs. FSC-H and SSC-W vs. SSC-H, viable cells were gated on, and of these, CD3 vs. CD14, $CD14^{-}CD3^{+}$ cells were gated on, and of these, $FOXP3^{+}$ cells were gated on, and of these, IFN-γ vs. SSC-A. Gate placement determined by FMO. Representative histogram with FMO.
For monocyte population; viable cells were gated on, and of these, $CD14^{+}$ and $CD3^{-}$ cells were gated upon; of these, SSC-A vs. IL-1β, IL-6, IL-12 identified. Gate placement for cytokines determined by FMO.

C) Significant reduction in IFN-γ production from $FOXP3^{+}$ Tregs of both patients with DAIH and HC subjects, co-cultured with silenced TLR 2 & 4 monocytes (DAIH Tregs: $p = 0.02$; $p = 0.02$ respectively; HC Tregs: $p = 0.02$; $p = 0.02$ respectively). Summary data: Top row: co-culture of monocytes with DAIH Tregs. Bottom row: co-culture of monocytes with HC Tregs. (sh – silenced, sc – scramble control).

D) $CD14^{++}$ monocytes with TLR 9 silenced fail to significantly reduce IFN-γ production from $FOXP3^{+}$ Tregs ($p = 0.2$). Summary data: Top row: co-culture of monocytes with DAIH Tregs. Bottom row: co-culture of monocytes with HC Tregs.

Fig. S4. $CD14^{++}$ monocytes from peripheral blood mononuclear cells (PBMC's) and $CD68^{+}$ macrophages from the liver of patients with DAIH display increased expression of inflammasome-associated components and absence of a negative regulator of infammasome Activation.
PBMC's from blood and intrahepatic lymphocytes from the liver were obtained from liver transplanted patients with DAIH (n=5), liver transplanted patients with normal allograft function who do not have DAIH (LTC) (n=4). PBMC's were also obtained from non- transplanted patients with autoimmune hepatitis (n=2), and stained for CD3CD14 (PBMC) and CD45CD68 (intrahepatic lymphocytes), and the monocyte ($CD3^{-}CD14^{++}$ PBMC) and macrophage ($CD45^{+}CD68^{+}$ liver) population were then sorted on a FACS Aria and subjected to library preparation for Single Cell Sequencing. After log normalizing and scaling the data, variable genes were detected using Seurat (*44*). These highly variable genes were used to generate principal components, and cluster detection was done using Seurat. The clusters were visualized using the tSNE rendering in Seurat.
A) There were13 individual cell clusters in the DAIH and LTC cohort of study subjects. Clusters 3 and 4 contain monocytes and macrophages of subjects with DAIH and cluster 6 contain monocytes and macrophages of LTC subjects. Red ovals highlight clusters 3 & 4, and cluster 6.

B) Cluster wise cell distribution confirmed that clusters 3 and 4 contain monocytes and macrophages of subjects with DAIH while cluster 6 contains monocytes and macrophages of LTC subjects. Red ovals highlight clusters 3 & 4, and cluster 6. Frequency represented on 'y' axis.

C) Cluster Specific Genes. Significantly over-expressed genes in monocytes and macrophages using single cell sequencing. A Venn diagram shows no overlap between significantly over-expressed

genes in clusters 3 + 4 vs. cluster 6 comparison, as well as in the cluster 6 vs. clusters 3 + 4 comparison.

D) Looking at the entire cohort of AIH, DAIH and LTC study subjects, there is 1 cluster of AIH cells that appears associated with the DAIH cluster of cells; additionally, some AIH cells intermingle with DAIH cells however this percentage is low.

E-F) Looking at the entire cohort of AIH, DAIH and LTC study subjects, Cluster wise cell distribution confirmed that cluster 3 has 90% proportion of AIH cells, 10% proportion of DAIH cells. Cluster 11 has 90% proportion of LTC cells. Frequency represented on 'y' axis.

G) After cluster detection, marker genes for each cluster were detected such that the gene(s) is expressed in at least 25% of the cells in the given cluster, and it is over expressed by at least 25% than all of the rest of the cells. The expression values for these genes were then plotted in each cell and rendered according to the tSNE to give the feature plots. Feature plots showing significant over expression of *TIMP1 (p = 9.0 X 10-223, 6 fold)* and *HSP90 (p = 1.1 x 10-95, fold > 3)* in cluster 3 compared to cluster 11 i.e. significant over expression in monocytes of subjects with AIH compared to monocytes/macrophages of LTC subjects.

Fig. S5. $CD14^{++}$ monocytes from patients with DAIH induce hepatocyte perturbation as evidenced by caspase-3 cleavage of hepatocytes.

$CD14^{++}$ monocytes from liver transplanted patients with DAIH (n=2), liver transplanted patients with normal allograft function who do not have DAIH (LTC) (n=2), and non transplanted children with AIH (n=2) were isolated by negative selection and co-cultured with normal hepatocytes over 96-hours, and anti-active caspase-3 production by hepatocytes were assessed by confocal microscopy.

A) Hepatocytes cultured alone for 24-hours with no added monocytes. No hepatocyte apoptosis observed. Top left: DAPI (nucleus) - grey, Top right: hepatocytes - green, Bottom left: caspase-3 – blue, Bottom right: merge. X100 magnification.

B) Hepatocytes cultured with monocytes from healthy subjects for 24-hours do not undergo hepatocyte apoptosis. Top left: DAPI (nucleus) - grey, Middle: hepatocytes - green, Top right: monocytes – orange, Bottom left: caspase-3 – blue, Middle: merge. X100 magnification.

C) Alanine aminotransferase (ALT) levels elevated in culture supernatant following 96-hours of co-culture of $CD14^{++}$ monocytes from subjects with de novo autoimmune hepatitis (DAIH) with normal hepatocytes (n = 2). Alanine aminotransferase levels not elevated in culture supernatant following 24-, 72- and 96-hours of co-culture of $CD14^{++}$ monocytes from: (i) transplanted subjects with normal allograft function who do not have DAIH (LTC) (n = 2), (ii) non-transplanted subjects with autoimmune hepatitis (AIH) (n = 2) and (iv) healthy, non-transplanted subjects (HC) (n = 2), with normal hepatocytes. Dotted line represents the upper limit of normal for ALT. Black bar: positive control. Hatched bar: DAIH.

Table S1. Single cell RNA-sequencing cell numbers.

Table S2. Serum alanine aminotransferase among subject groups.

Table S3. Correlation between release of DAMPs and serum alanine aminotransferase level in subjects with DAIH.

Table S4. Correlation between release of DAMPs and serum alanine aminotransferase level in subjects with AIH.

2 Supplementary Figures and Tables

For more information on Supplementary Material and for details on the different file types accepted, please see here.

2.1 Supplementary Figures

Fig. S1a.

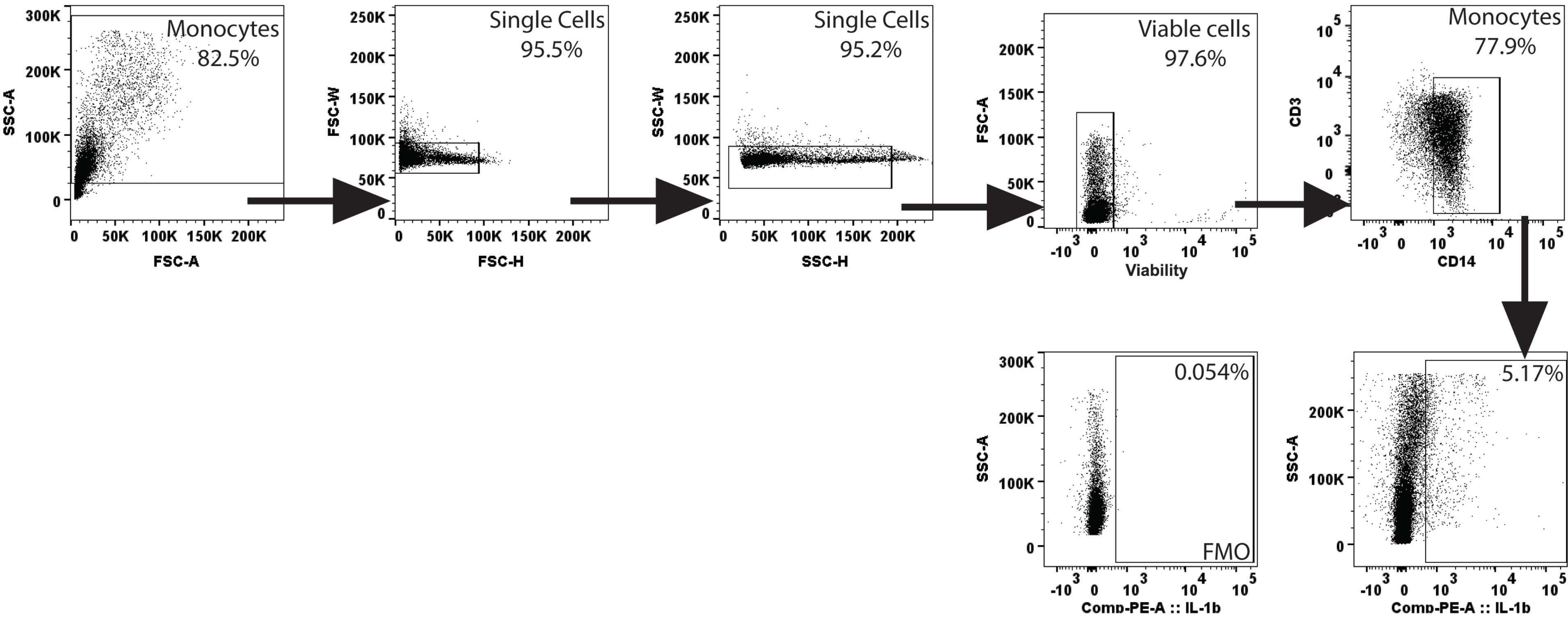
Monocytes
82.5%
SSC-A
FSC-A
Single Cells
95.5%
FSC-W
FSC-H
Single Cells
95.2%
SSC-W
SSC-H
Viable cells
97.6%
FSC-A
Viability
Monocytes
77.9%
CD3
CD14
0.054%
FMO
SSC-A
Comp-PE-A :: IL-1b
5.17%
SSC-A
Comp-PE-A :: IL-1b

Fig. S1b.

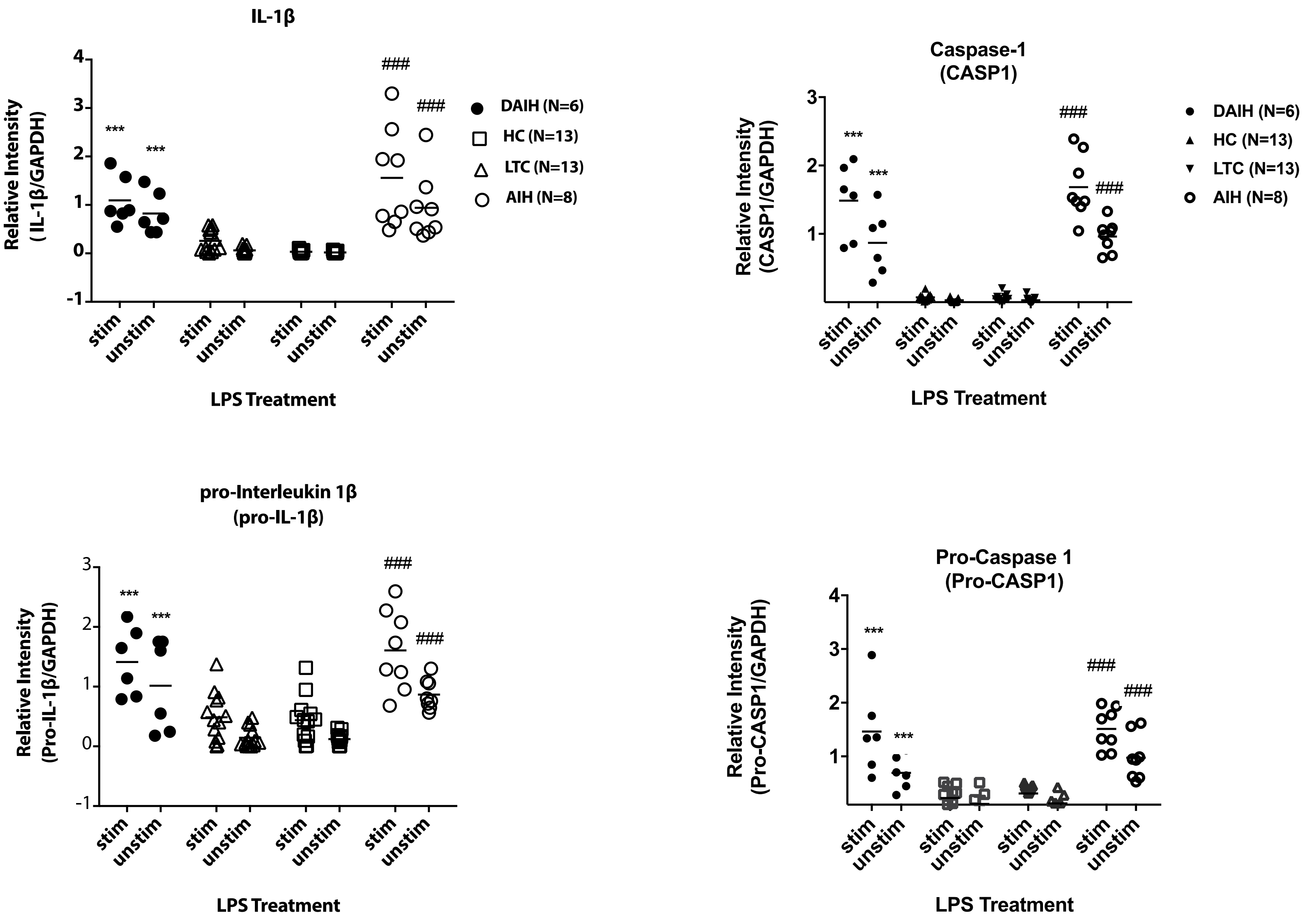
IL-1β
Relative Intensity
(IL-1β/GAPDH)
4
3
2
1
0
-1
***
***
###
###
stim
unstim
stim
unstim
stim
unstim
stim
unstim
LPS Treatment
DAIH (N=6)
HC (N=13)
LTC (N=13)
AIH (N=8)
Caspase-1
(CASP1)
Relative Intensity
(CASP1/GAPDH)
3
2
1
***
***
###
###
stim
unstim
stim
unstim
stim
unstim
stim
unstim
LPS Treatment
DAIH (N=6)
HC (N=13)
LTC (N=13)
AIH (N=8)
pro-Interleukin 1β
(pro-IL-1β)
Relative Intensity
(Pro-IL-1β/GAPDH)
3
2
1
0
-1
***
***
###
###
stim
unstim
stim
unstim
stim
unstim
stim
unstim
LPS Treatment
Pro-Caspase 1
(Pro-CASP1)
Relative Intensity
(Pro-CASP1/GAPDH)
4
3
2
1
***
***
###
###
stim
unstim
stim
unstim
stim
unstim
stim
unstim
LPS Treatment

Fig. S1b.

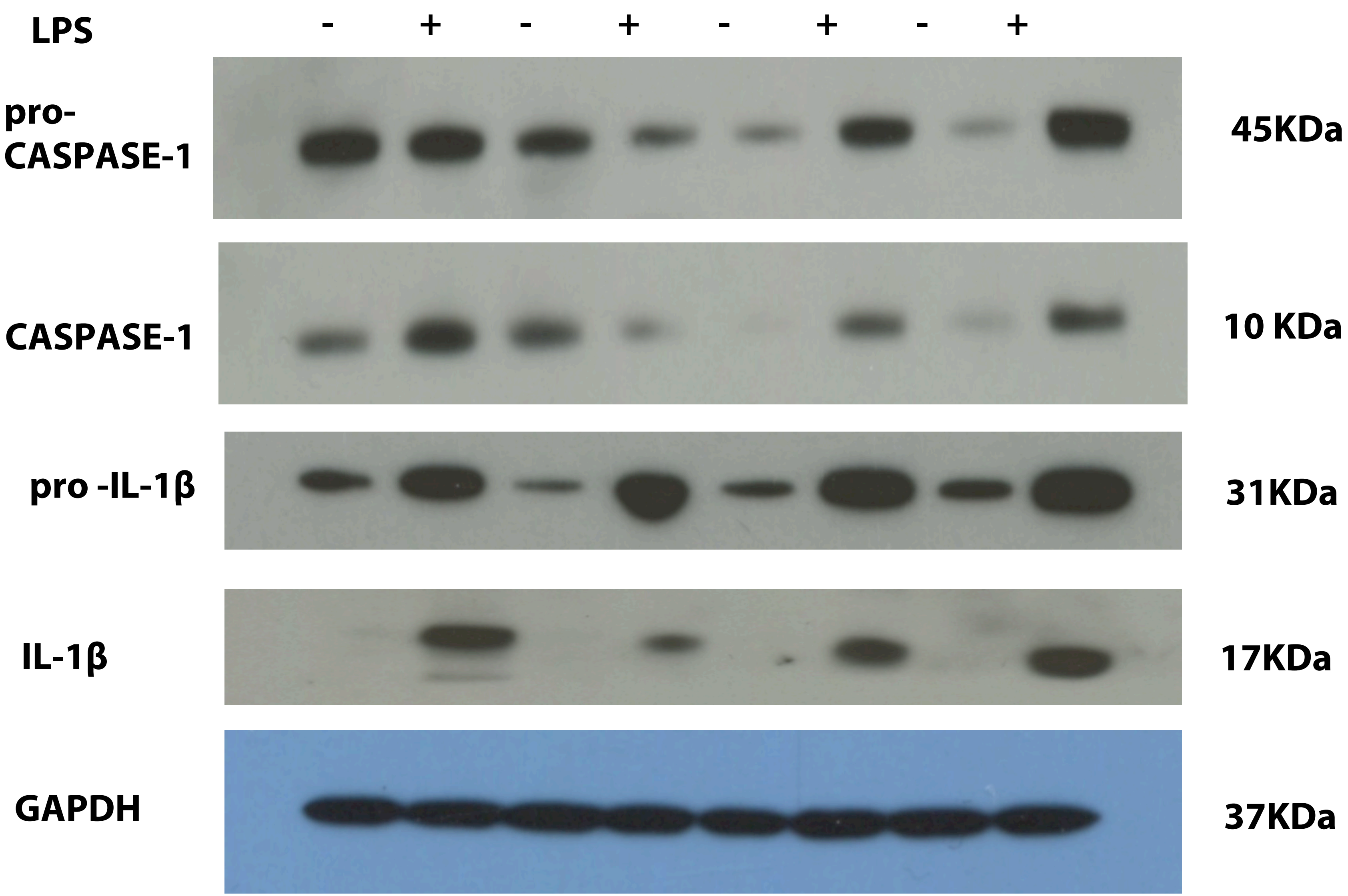
LPS
- + - + - + - +
pro-CASPASE-1
45KDa
CASPASE-1
10 KDa
pro -IL-1β
31KDa
IL-1β
17KDa
GAPDH
37KDa

Fig. S1b.

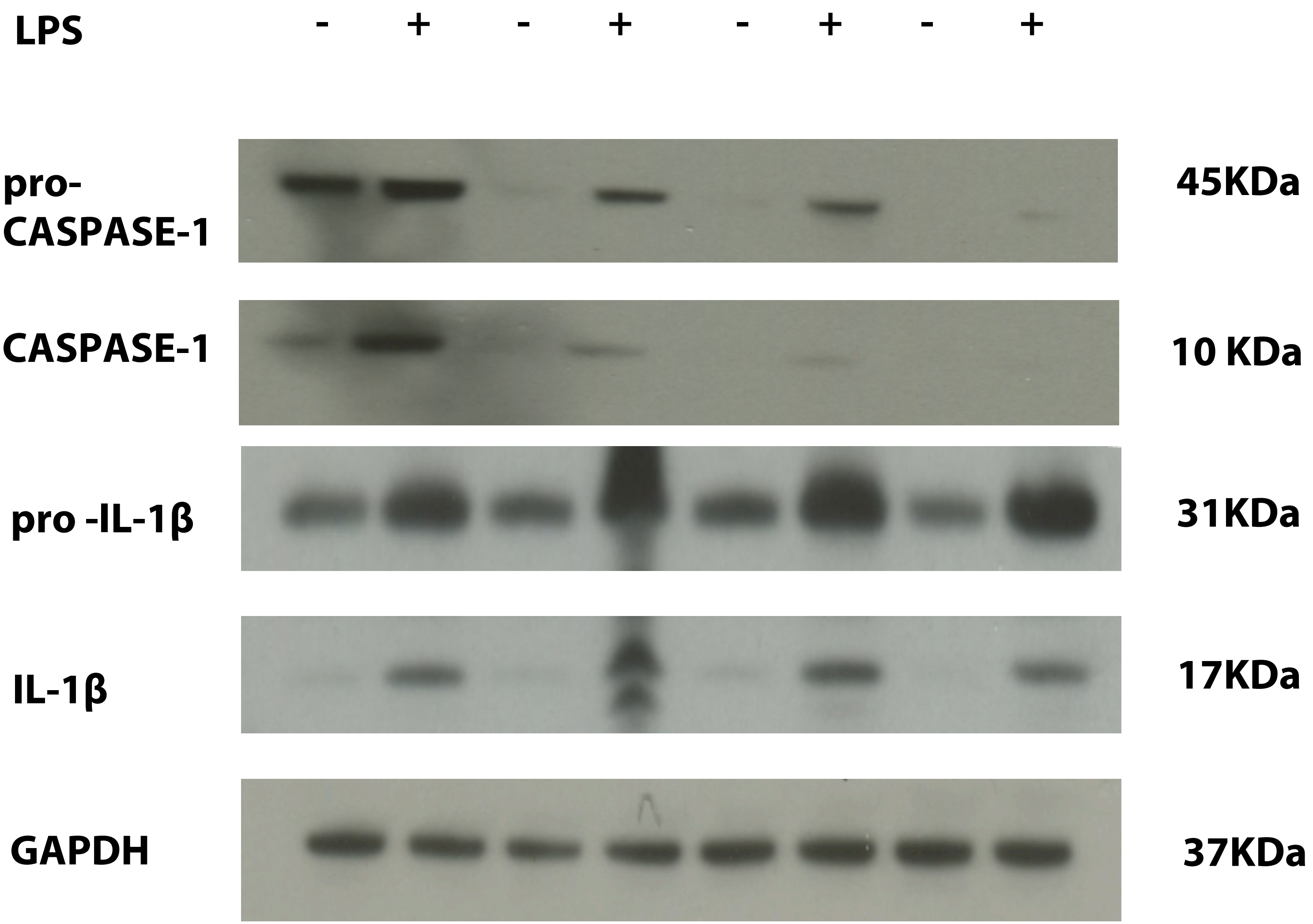
LPS
- + - + - + - +
pro-
CASPASE-1
45KDa
CASPASE-1
10 KDa
pro -IL-1β
31KDa
IL-1β
17KDa
GAPDH
37KDa

Fig. S1c. Representative plates: protein Inhibition targeting heat shock protein, fibrinogen, & complex I –III.

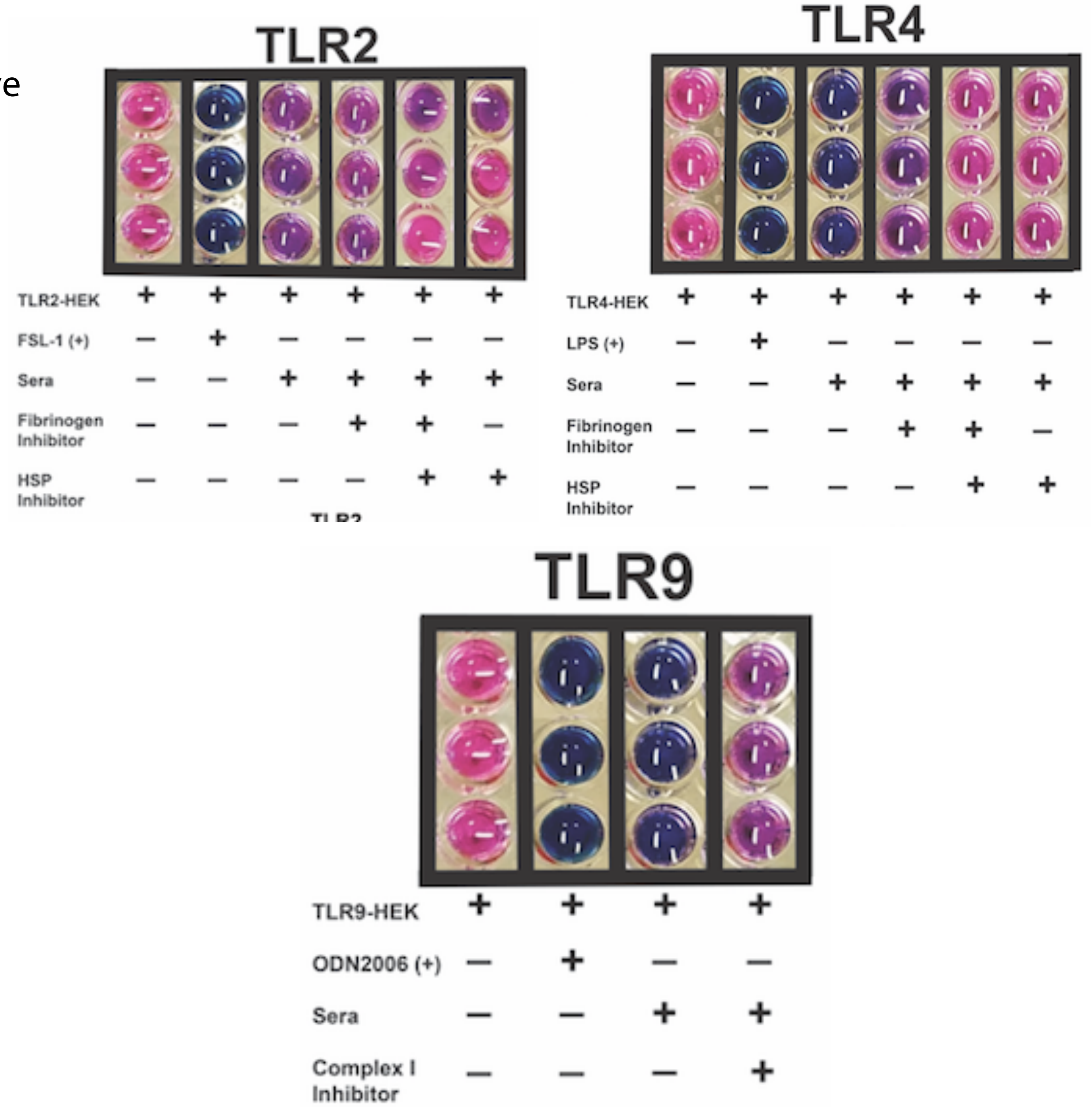

Fig. S1d.

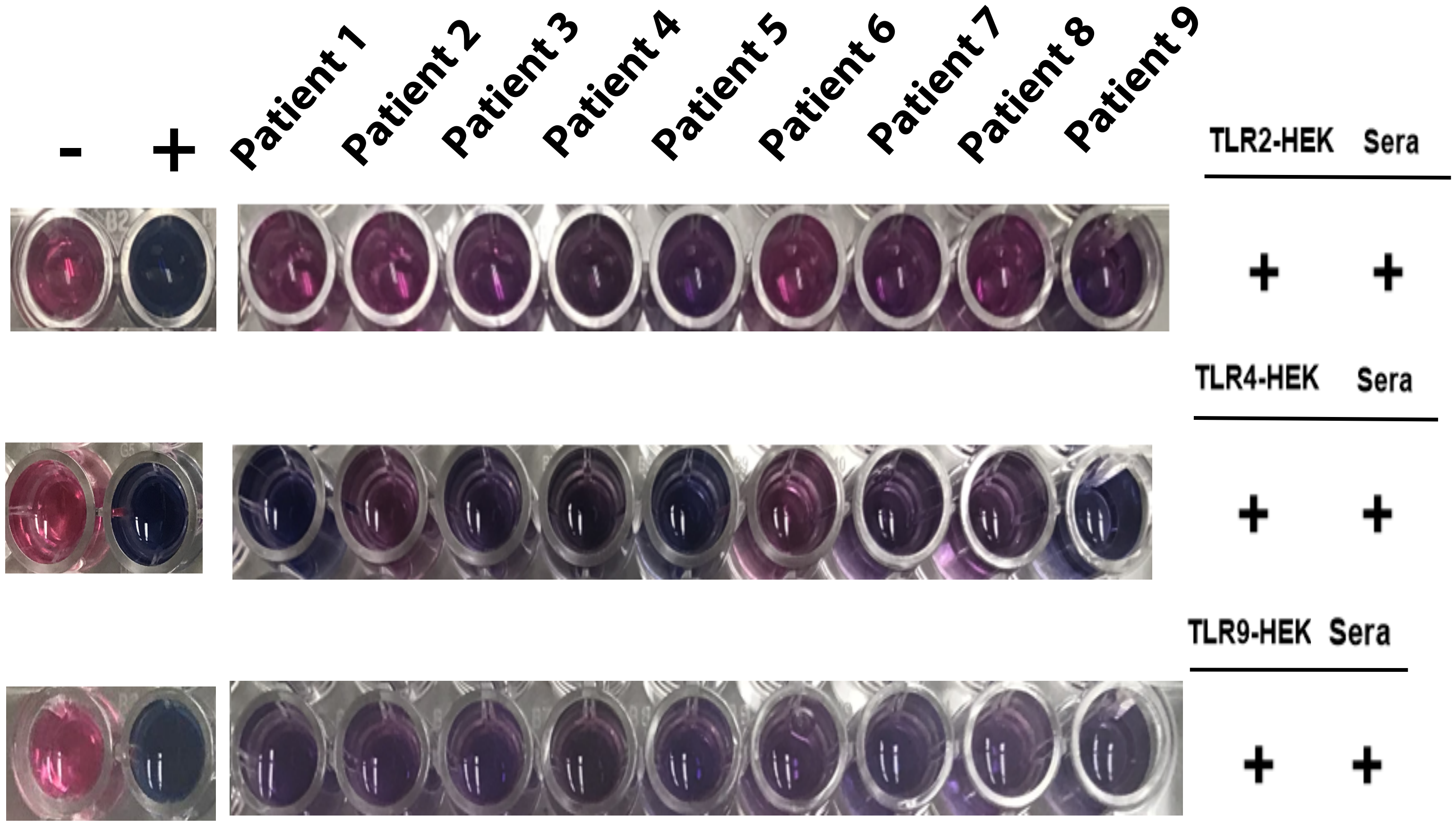
-
+
Patient 1
Patient 2
Patient 3
Patient 4
Patient 5
Patient 6
Patient 7
Patient 8
Patient 9
TLR2-HEK Sera
+ +
TLR4-HEK Sera
+ +
TLR9-HEK Sera
+ +

Fig. S1d.

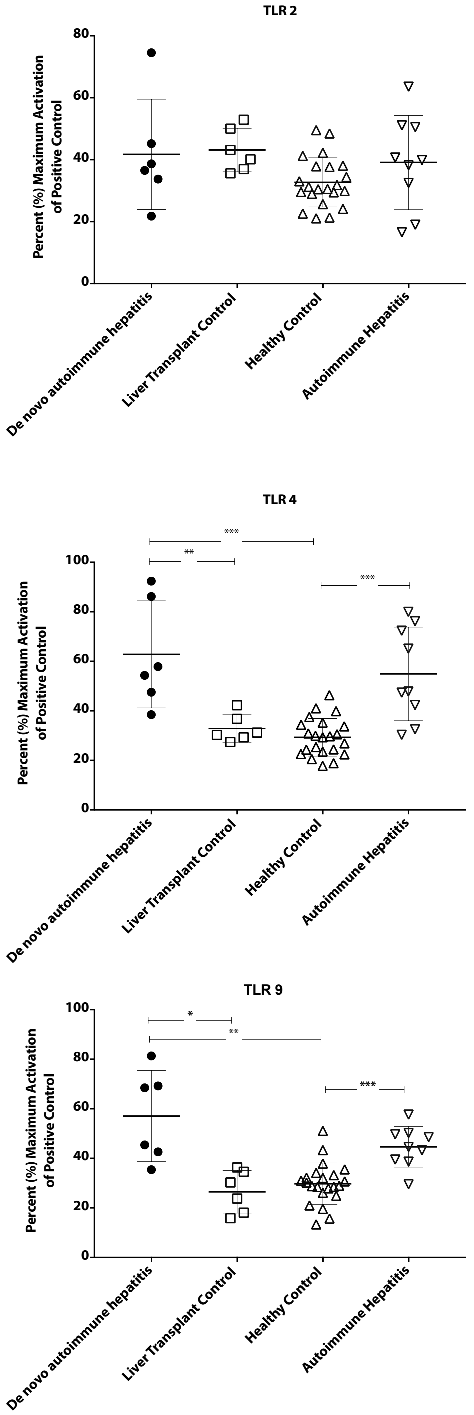
TLR 2
Percent (%) Maximum Activation of Positive Control
80
60
40
20
0
De novo autoimmune hepatitis
Liver Transplant Control
Healthy Control
Autoimmune Hepatitis
TLR 4
***
**
***
100
80
60
40
20
0
TLR 9
*
**
***

Fig. S1e. ALT negatively correlated with mitochondrial DNA in subjects with AIH.

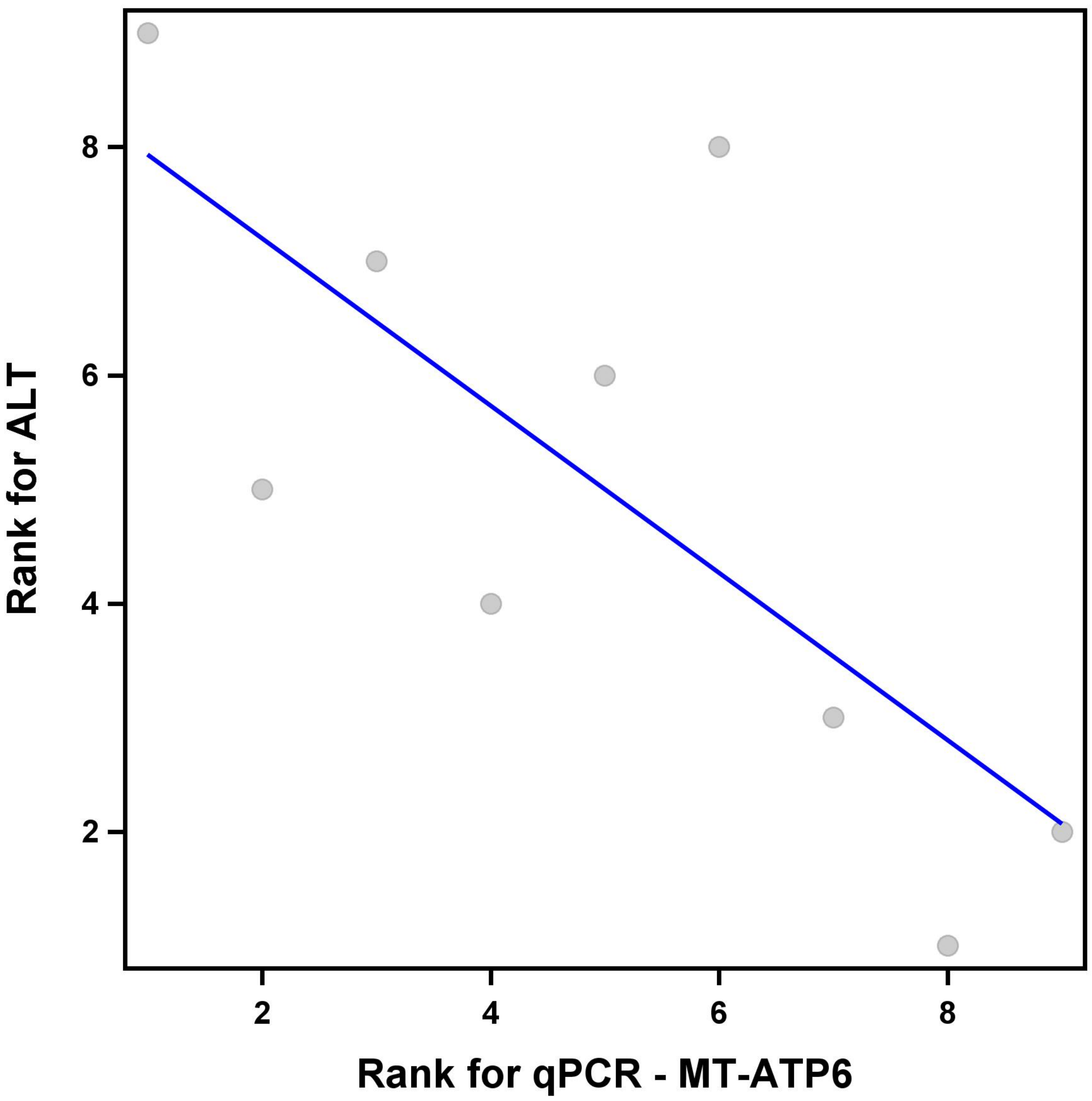

Fig. S2a - b.

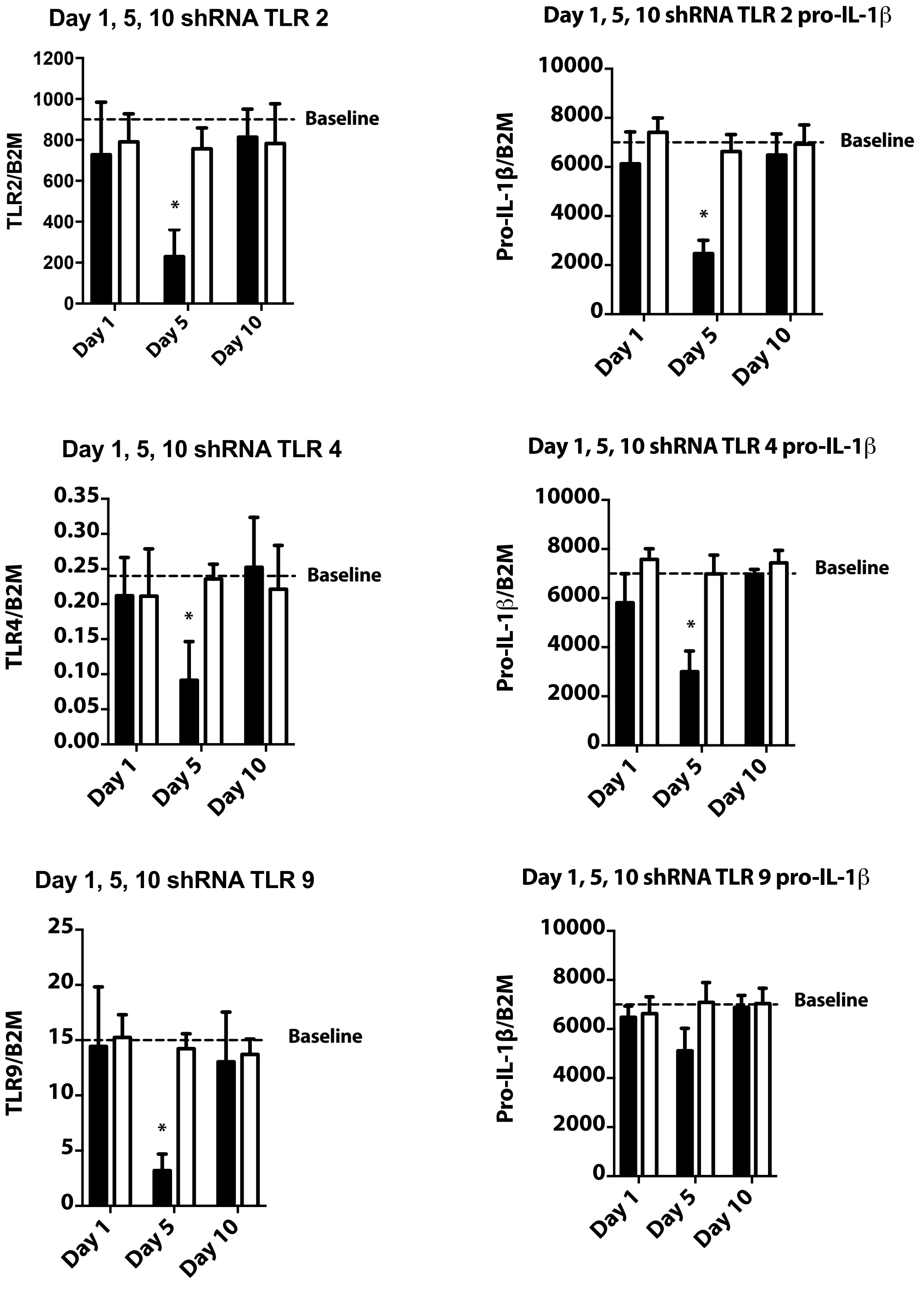

Day 1, 5, 10 shRNA TLR 2
TLR2/B2M
1200
1000
800
600
400
200
0
Baseline
*
Day 1
Day 5
Day 10
Day 1, 5, 10 shRNA TLR 2 pro-IL-1β
Pro-IL-1β/B2M
10000
8000
6000
4000
2000
0
Baseline
*
Day 1
Day 5
Day 10
Day 1, 5, 10 shRNA TLR 4
TLR4/B2M
0.35
0.30
0.25
0.20
0.15
0.10
0.05
0.00
Baseline
*
Day 1
Day 5
Day 10
Day 1, 5, 10 shRNA TLR 4 pro-IL-1β
Pro-IL-1β/B2M
10000
8000
6000
4000
2000
0
Baseline
*
Day 1
Day 5
Day 10
Day 1, 5, 10 shRNA TLR 9
TLR9/B2M
25
20
15
10
5
0
Baseline
*
Day 1
Day 5
Day 10
Day 1, 5, 10 shRNA TLR 9 pro-IL-1β
Pro-IL-1β/B2M
10000
8000
6000
4000
2000
0
Baseline
Day 1
Day 5
Day 10

Fig. S2c.

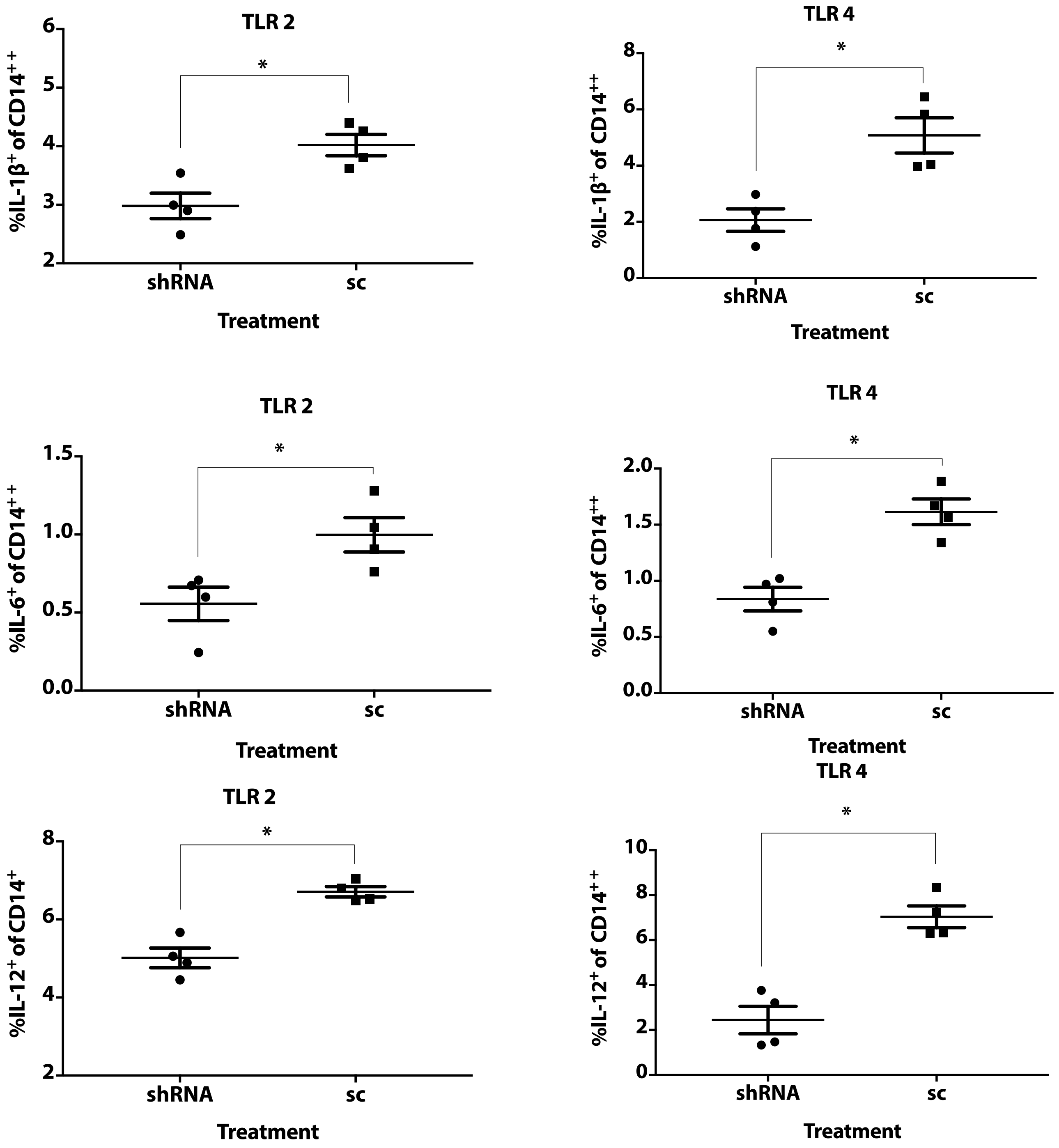
TLR 2
%IL-1β+ of CD14++
6
5
4
3
2
*
shRNA
sc
Treatment
TLR 4
%IL-1β+ of CD14++
8
6
4
2
0
*
shRNA
sc
Treatment
TLR 2
%IL-6+ of CD14++
1.5
1.0
0.5
0.0
*
shRNA
sc
Treatment
TLR 4
%IL-6+ of CD14++
2.0
1.5
1.0
0.5
0.0
*
shRNA
sc
Treatment
TLR 2
%IL-12+ of CD14++
8
6
4
2
*
shRNA
sc
Treatment
TLR 4
%IL-12+ of CD14++
10
8
6
4
2
0
*
shRNA
sc
Treatment

Fig. S2c.

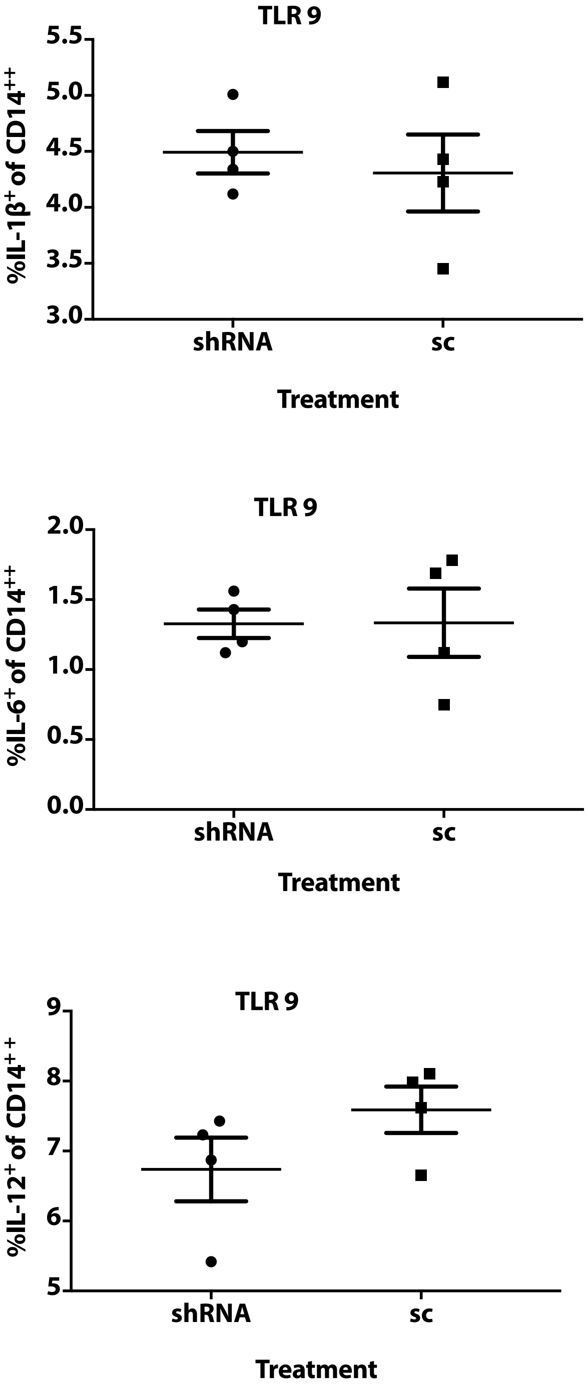
TLR 9
%IL-1β+ of CD14++
5.5
5.0
4.5
4.0
3.5
3.0
shRNA
sc
Treatment
TLR 9
%IL-6+ of CD14++
2.0
1.5
1.0
0.5
0.0
shRNA
sc
Treatment
TLR 9
%IL-12+ of CD14++
9
8
7
6
5
shRNA
sc
Treatment

Fig. S2d.

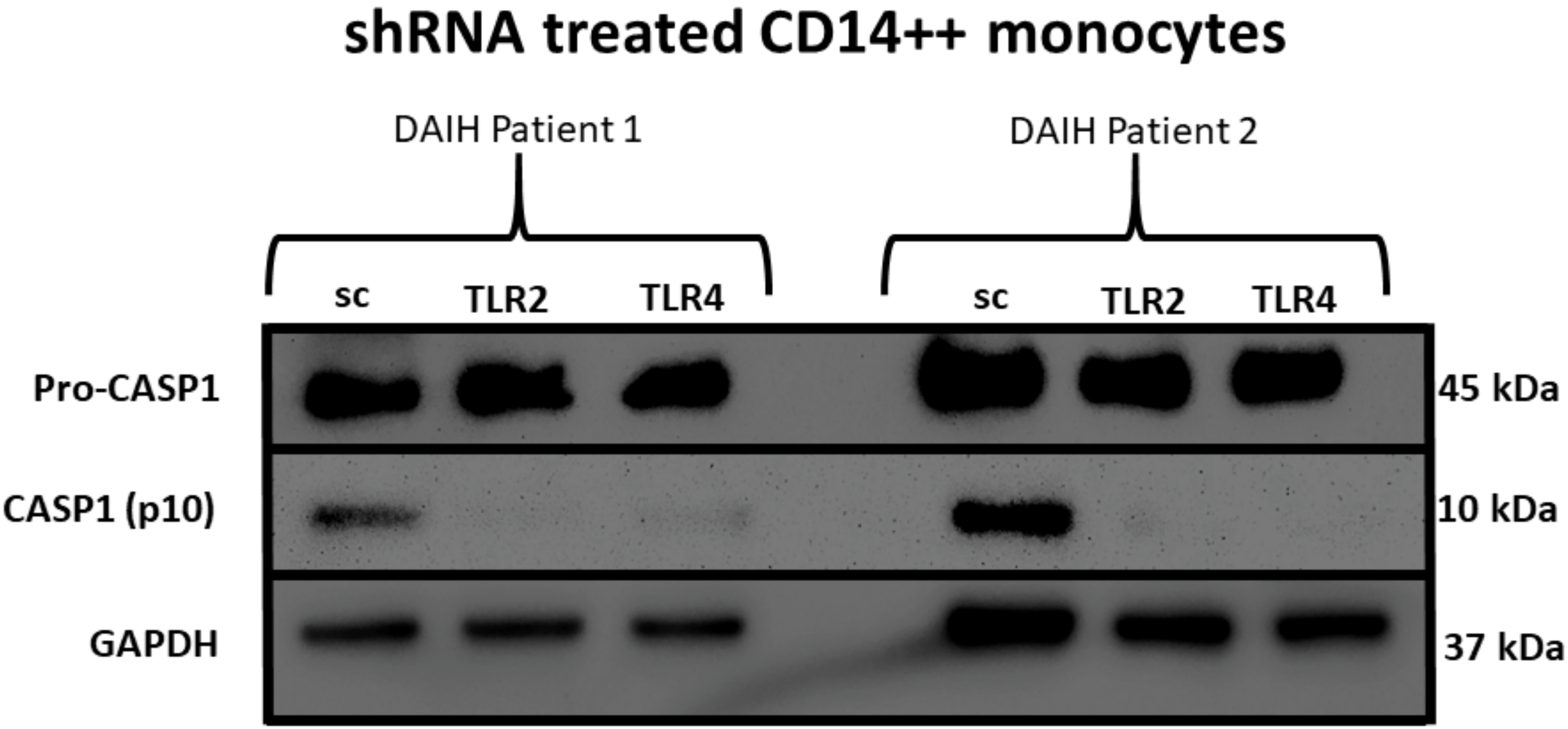
shRNA treated CD14++ monocytes
DAIH Patient 1
DAIH Patient 2
sc
TLR2
TLR4
sc
TLR2
TLR4
Pro-CASP1
CASP1 (p10)
GAPDH
45 kDa
10 kDa
37 kDa

Fig. S2d.

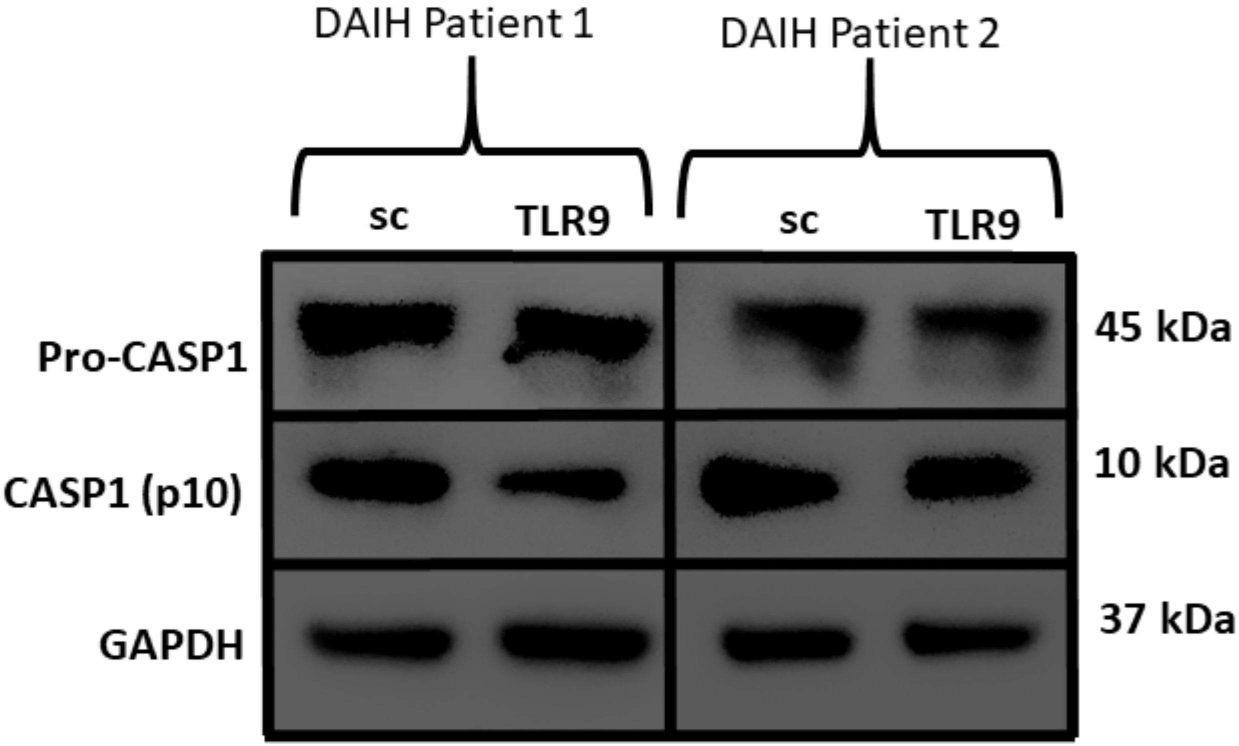
DAIH Patient 1
DAIH Patient 2
sc
TLR9
sc
TLR9
Pro-CASP1
CASP1 (p10)
GAPDH
45 kDa
10 kDa
37 kDa

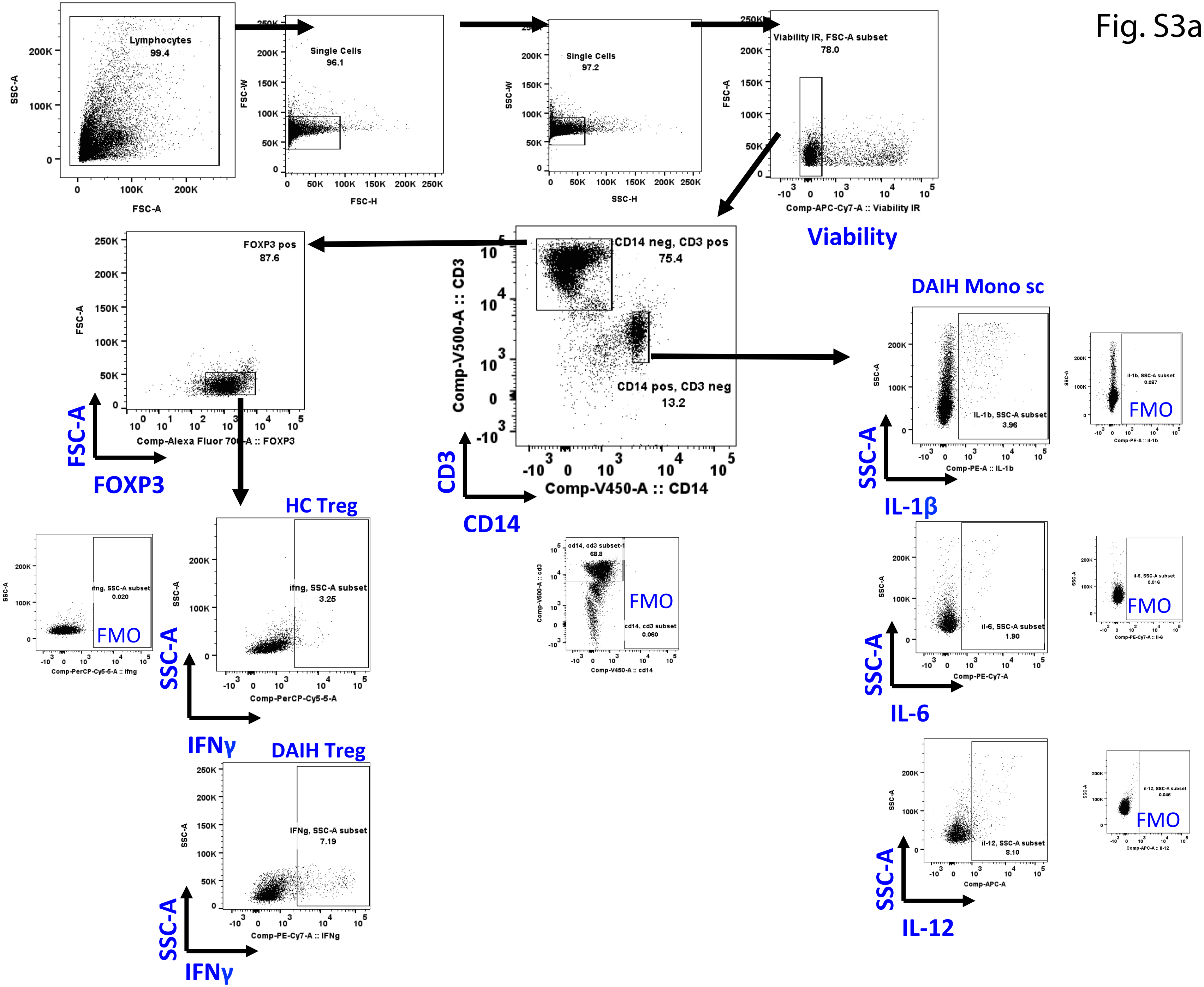
Lymphocytes
99.4
SSC-A
FSC-A
Single Cells
96.1
FSC-W
FSC-H
Single Cells
97.2
SSC-W
SSC-H
Viability IR, FSC-A subset
78.0
FSC-A
Comp-APC-Cy7-A :: Viability IR
Viability
CD14 neg, CD3 pos
75.4
CD14 pos, CD3 neg
13.2
Comp-V500-A :: CD3
Comp-V450-A :: CD14
CD3
CD14
FOXP3 pos
87.6
FSC-A
Comp-Alexa Fluor 700-A :: FOXP3
FOXP3
DAIH Mono sc
IL-1b, SSC-A subset
3.96
Comp-PE-A :: IL-1b
il-1b, SSC-A subset
0.087
FMO
SSC-A
IL-1β
HC Treg
ifng, SSC-A subset
3.25
Comp-PerCP-Cy5-5-A
ifng, SSC-A subset
0.020
Comp-PerCP-Cy5-5-A :: ifng
FMO
SSC-A
IFNγ
cd14, cd3 subset-1
68.8
cd14, cd3 subset
0.060
Comp-V500-A :: cd3
Comp-V450-A :: cd14
FMO
il-6, SSC-A subset
1.90
Comp-PE-Cy7-A
il-6, SSC-A subset
0.016
Comp-PE-Cy7-A :: il-6
FMO
SSC-A
IL-6
DAIH Treg
IFNg, SSC-A subset
7.19
Comp-PE-Cy7-A :: IFNg
SSC-A
IFNγ
il-12, SSC-A subset
8.10
Comp-APC-A
il-12, SSC-A subset
0.045
Comp-APC-A :: il-12
FMO
SSC-A
IL-12

Fig. S3a.

Fig. S3b.

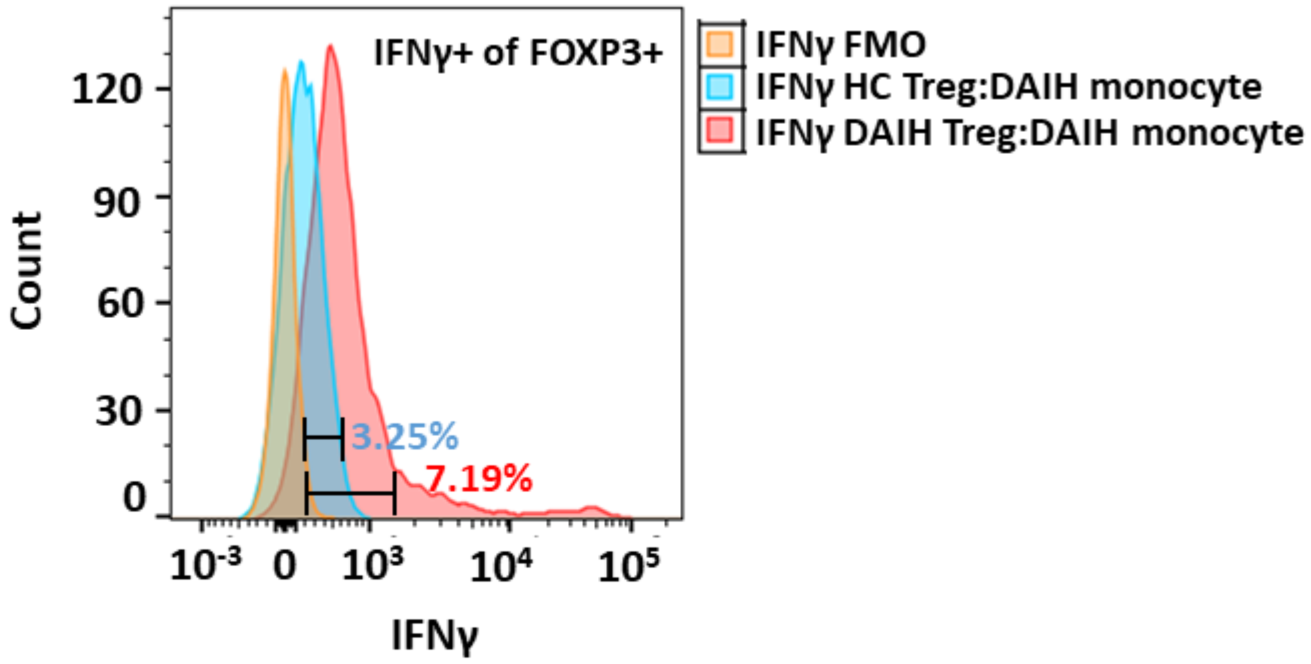
IFNγ+ of FOXP3+
IFNγ FMO
IFNγ HC Treg:DAIH monocyte
IFNγ DAIH Treg:DAIH monocyte
120
90
60
30
0
Count
3.25%
7.19%
10-3
0
103
104
105
IFNγ

Fig. S3c. Summary data: silencing of TLRs 2 & 4 in DAIH monocytes. Top row: DAIH monocytes + DAIH Tregs. Bottom row: DAIH monocytes + HC Tregs.

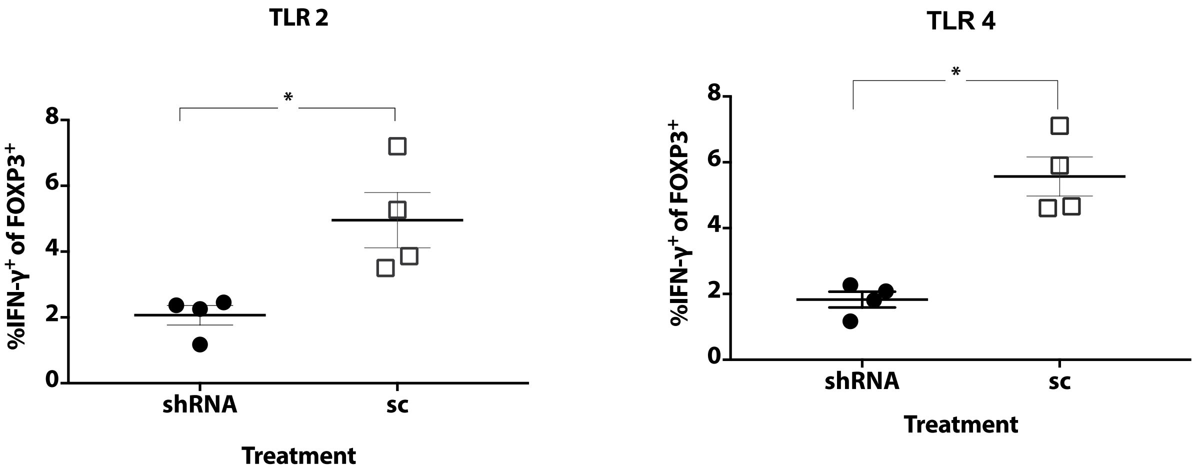

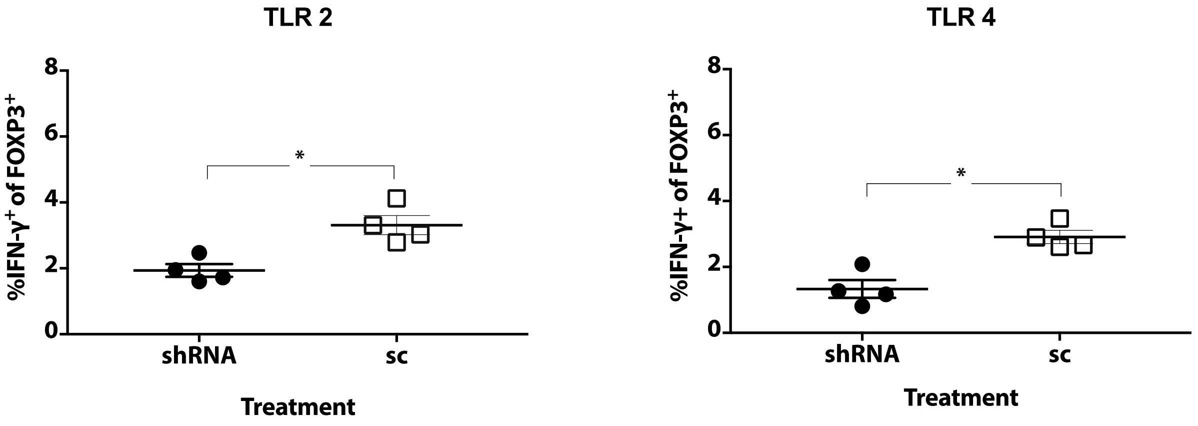

Fig. S3d. Summary data: silencing of TLR 9 in DAIH monocytes. Top row: DAIH monocytes + DAIH Tregs. Bottom row: DAIH monocytes + HC Tregs.

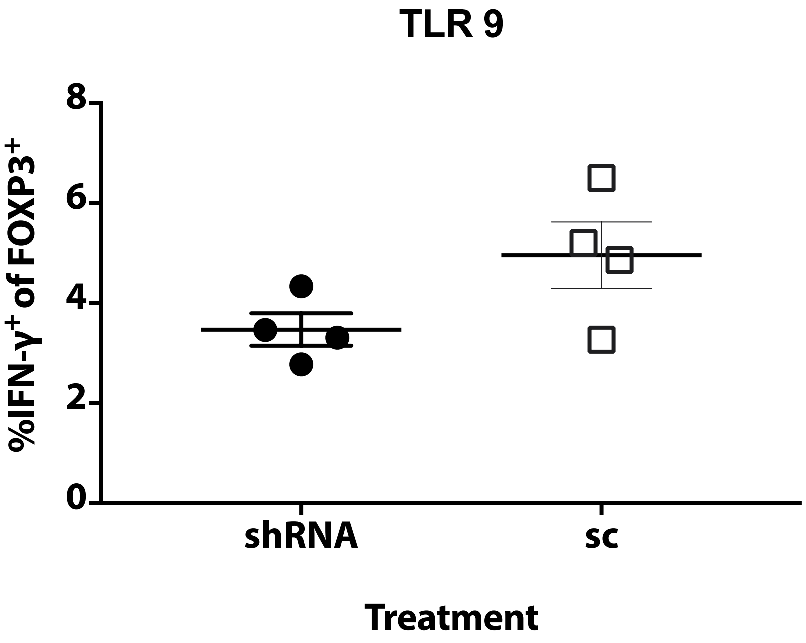

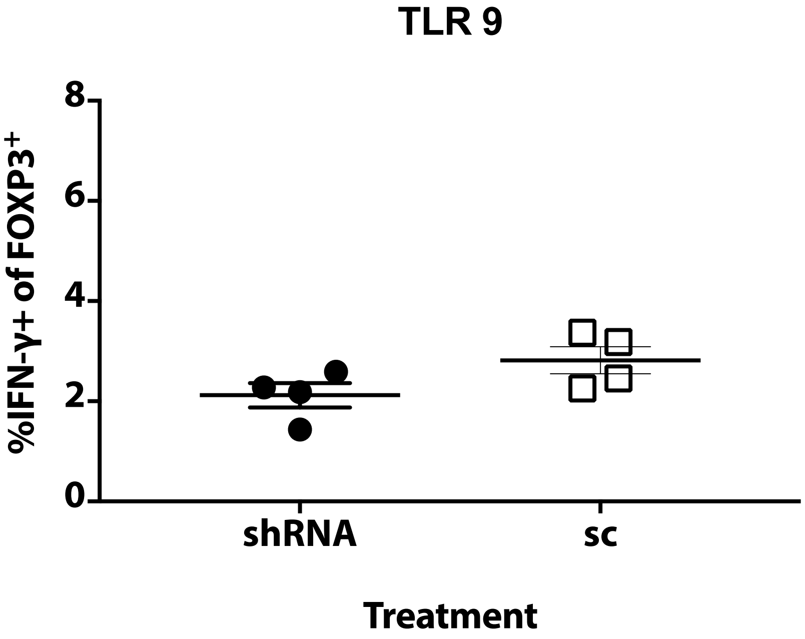

Fig. S4a.
Cells in clusters 3 & 4 = DAIH macrophages/ monocytes.
Cells in cluster 6 = LTC macrophages/ monocytes.

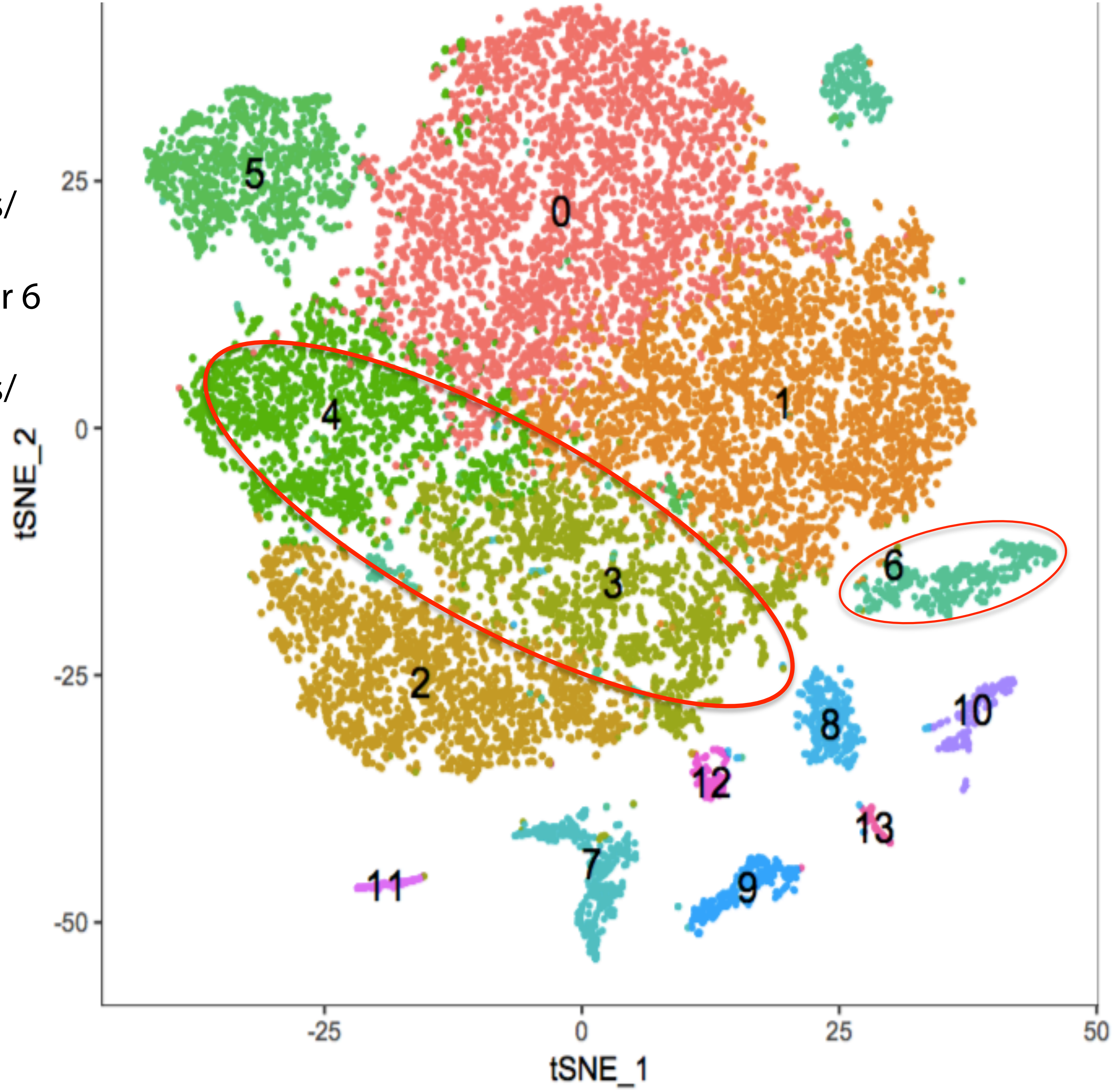

Fig. S4b. Clusterwise Cell Distribution

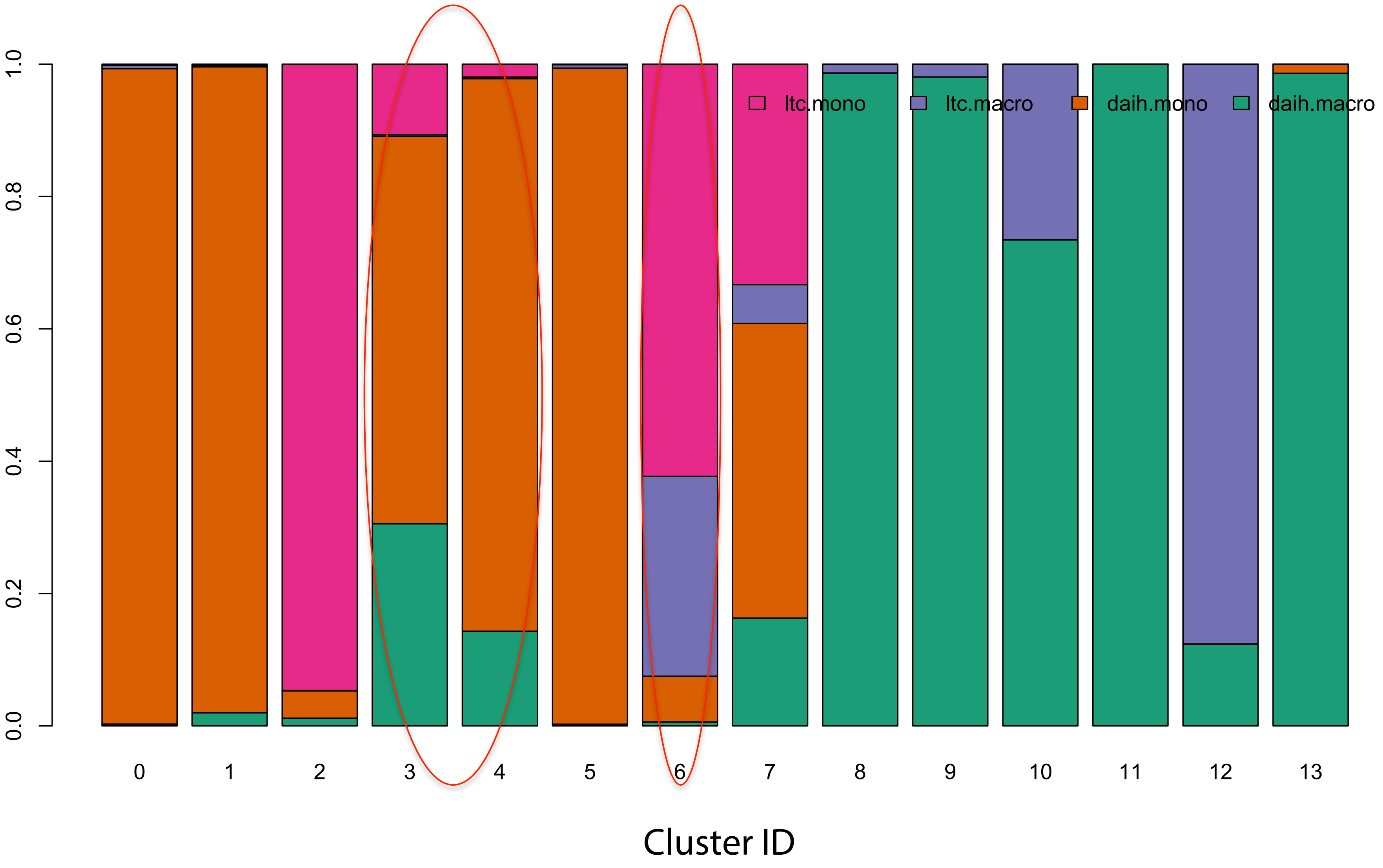

Cluster Specific Genes

Fig. S4c.
Comparison of cells in clusters 3 & 4 vs. cluster 6 shows no overlap in significantly expressed genes.

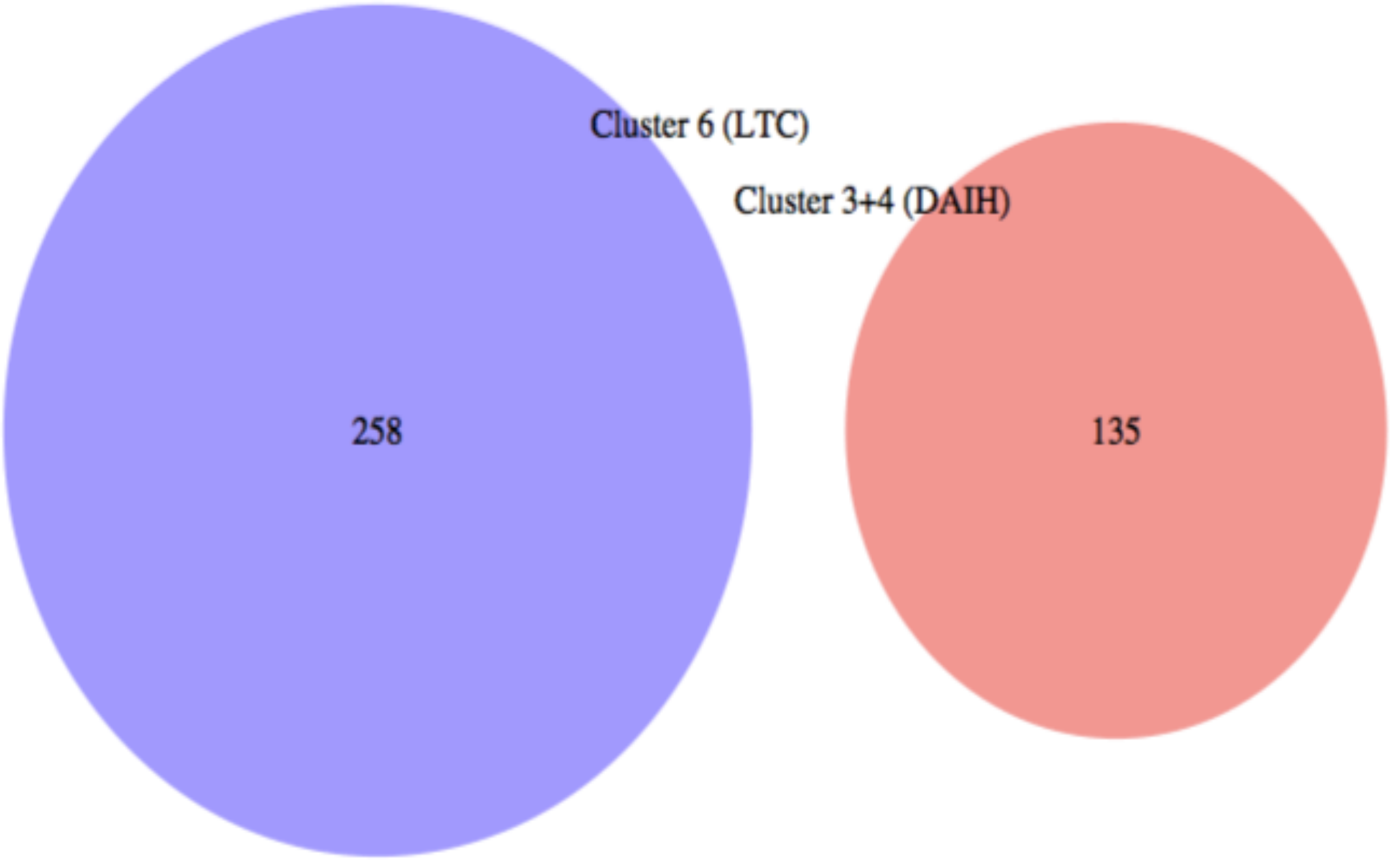

Fig. S4d.
1 AIH cluster appears associated with the DAIH cluster of cells and some AIH cells intermingle with DAIH cells but this percentage is low.

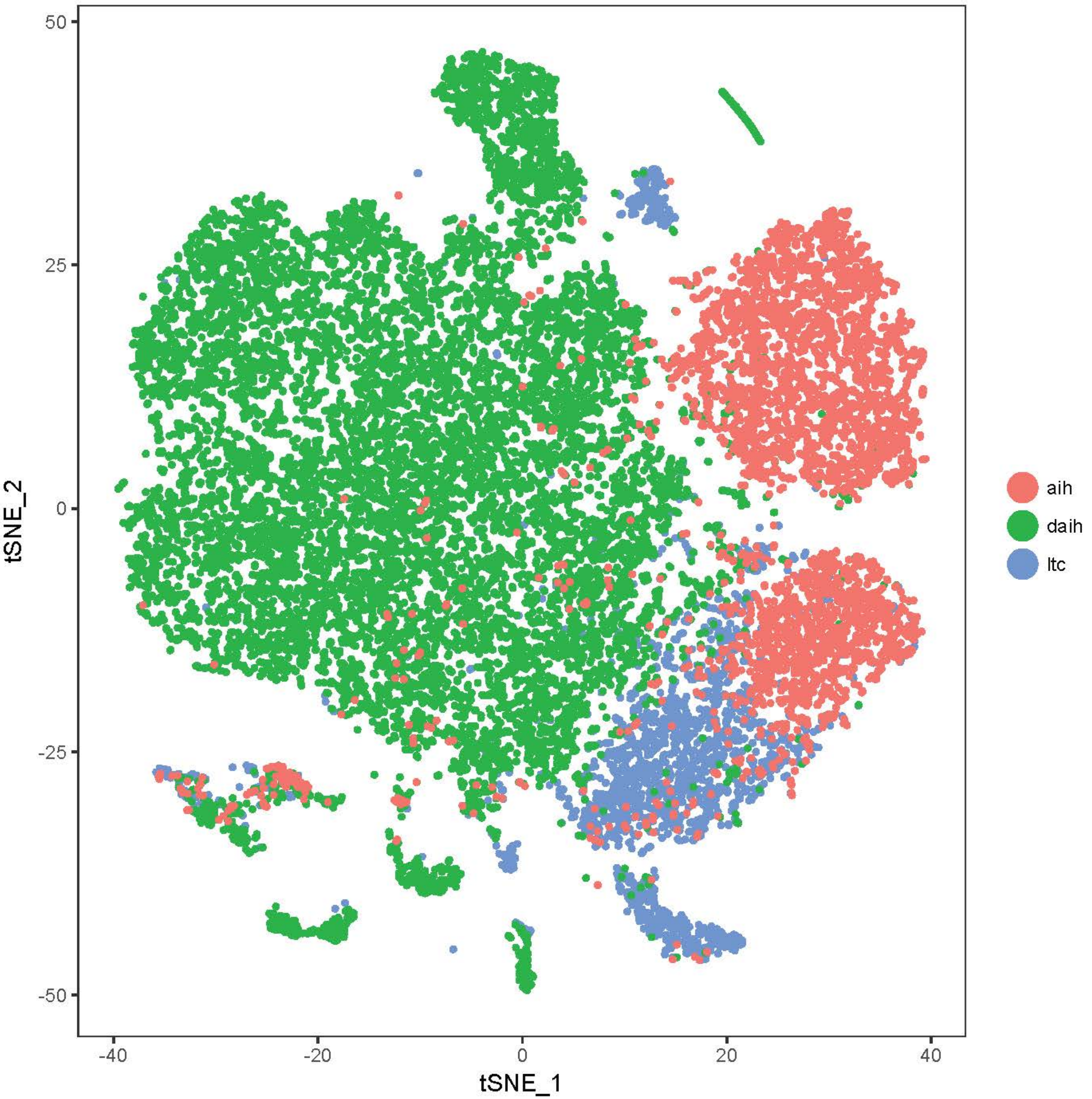

Fig. S4e. Cells in clusters 3 = AIH monocytes. Cells in cluster 11 = LTC macrophages/ monocytes.

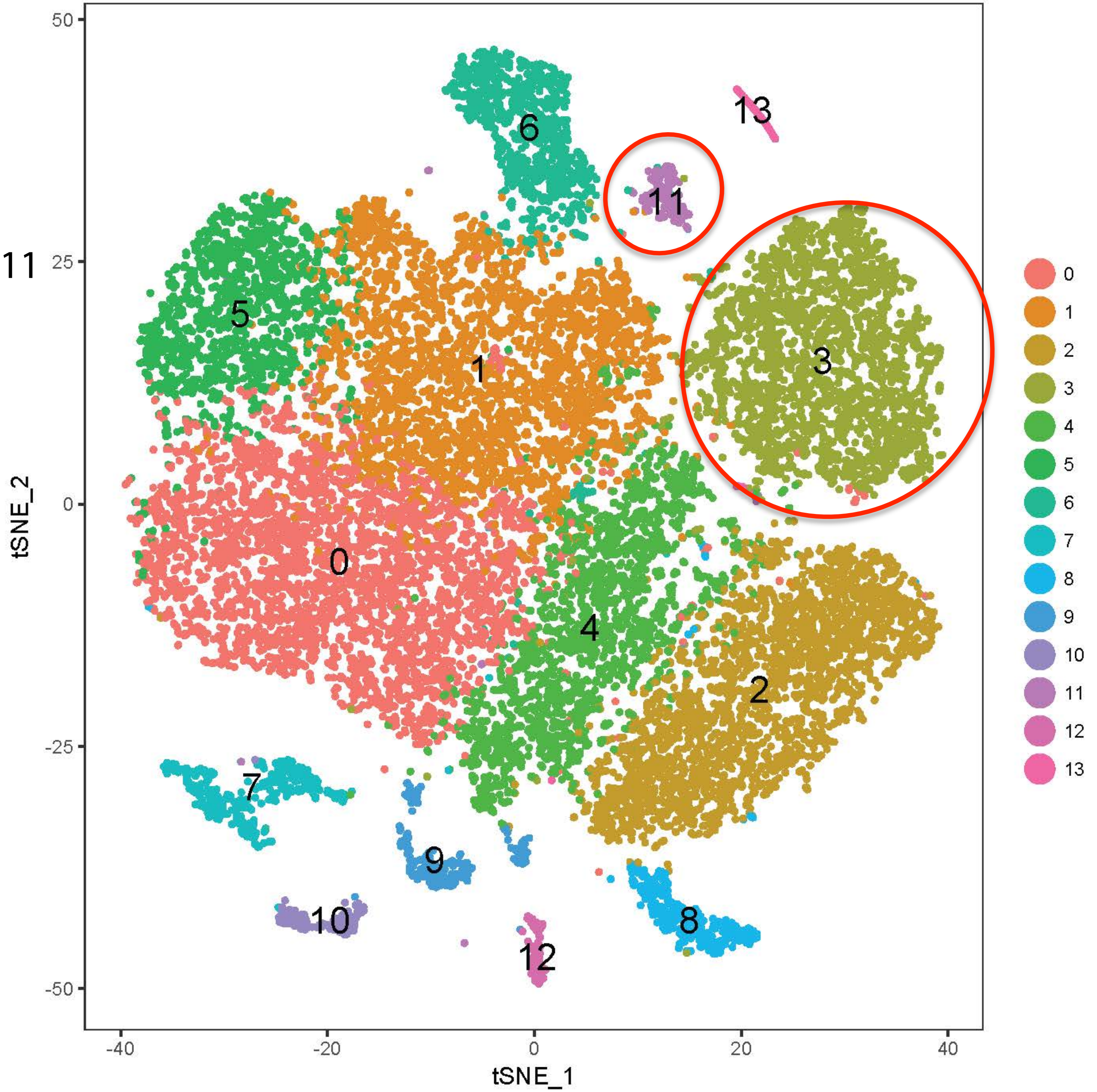

Fig. S4f. Clusterwise Cell Distribution
Cluster 3 has 90% proportion of AIH cells and 10% proportion of DAIH cells.
Cluster 11 has 90% proportion of LTC cells.

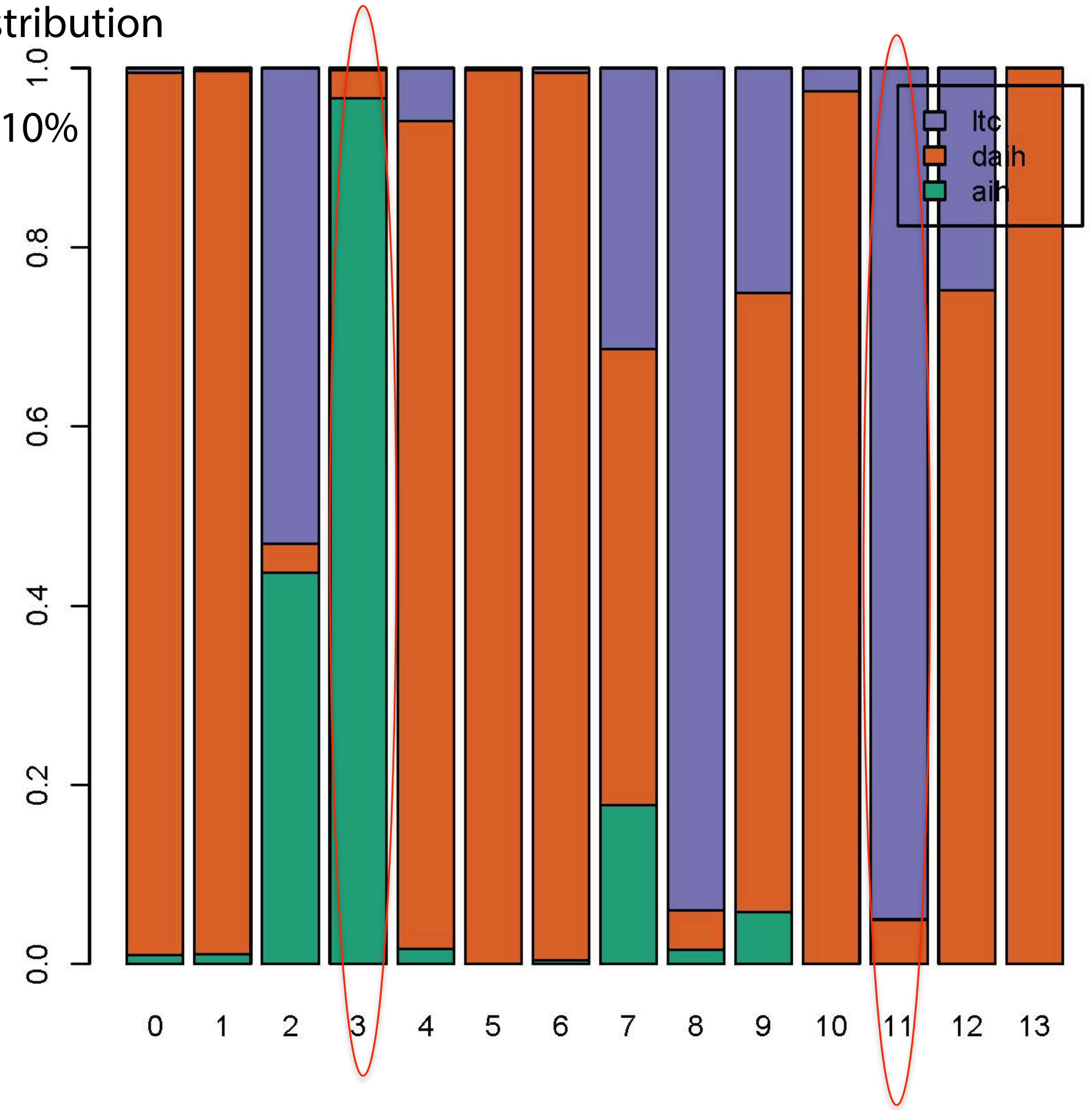

Fig. S4g. Significant over expression of *TIMP1* and *HSP90* in AIH monocytes compared to LTC macrophages/monocytes.

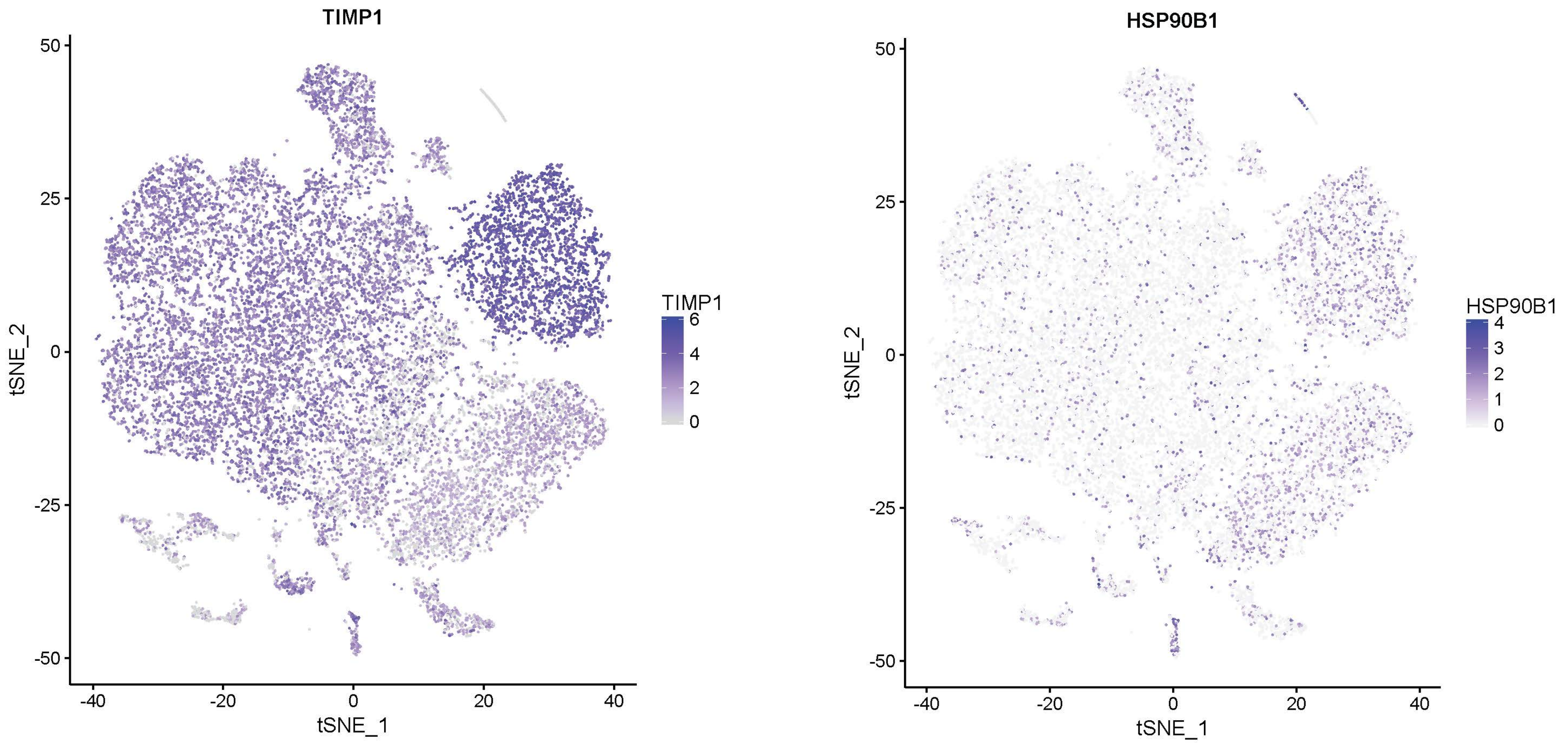

Fig. S5a.

Hepatocytes alone

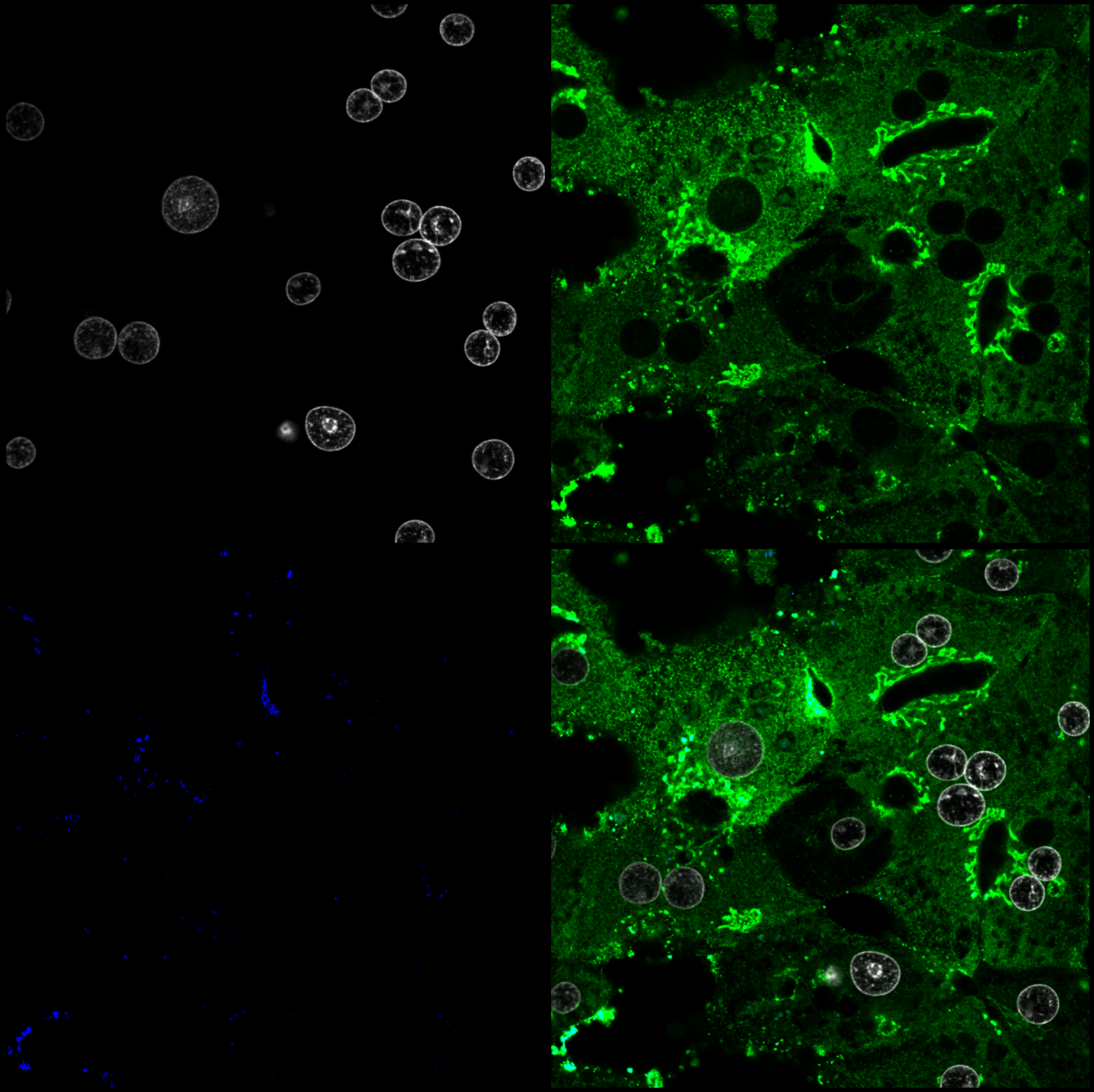

DAPI (nucleus)
Hepatocyte
Caspase-3

X100

Merge

Fig. S5b.

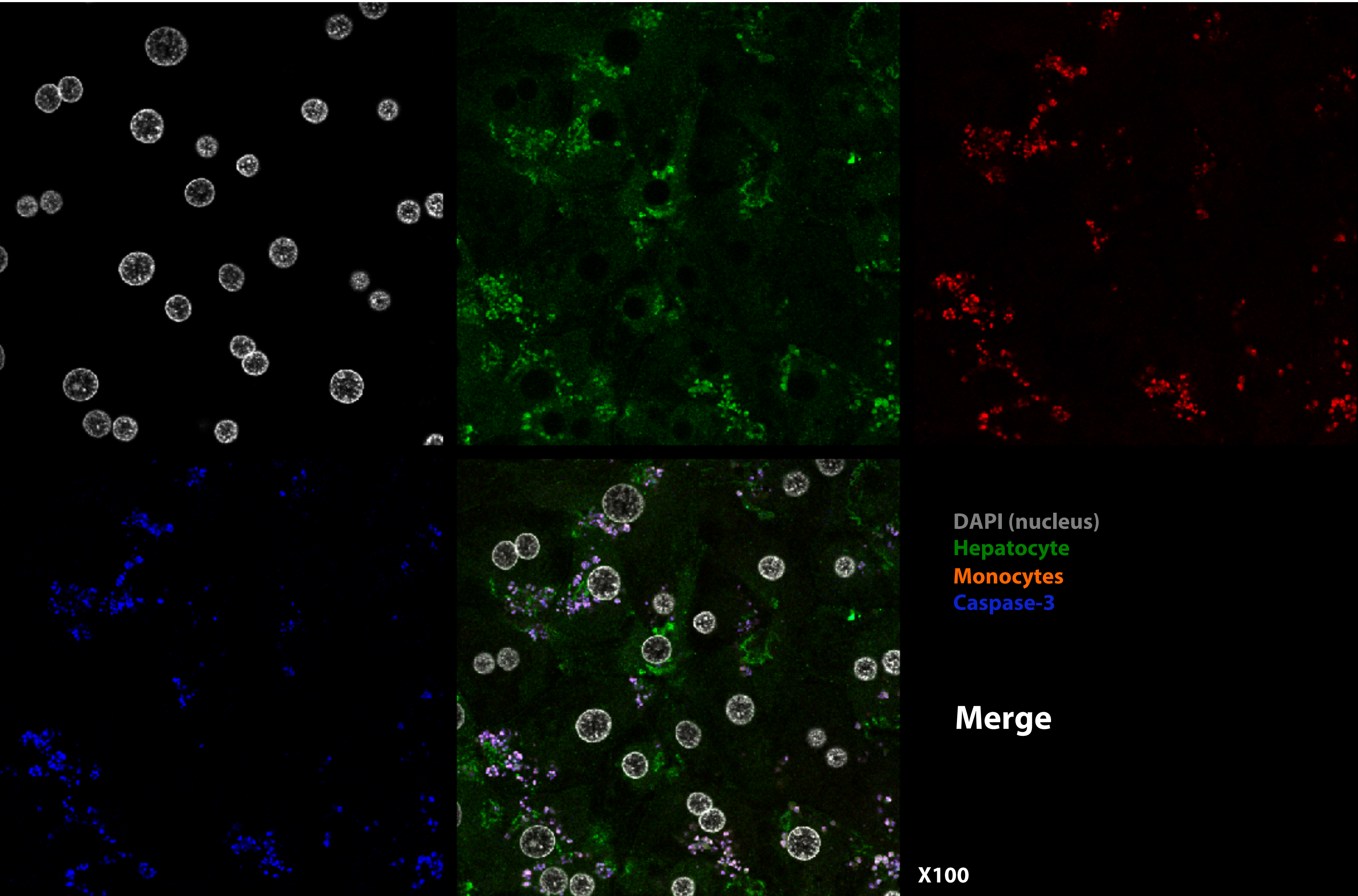

Fig. S5c.

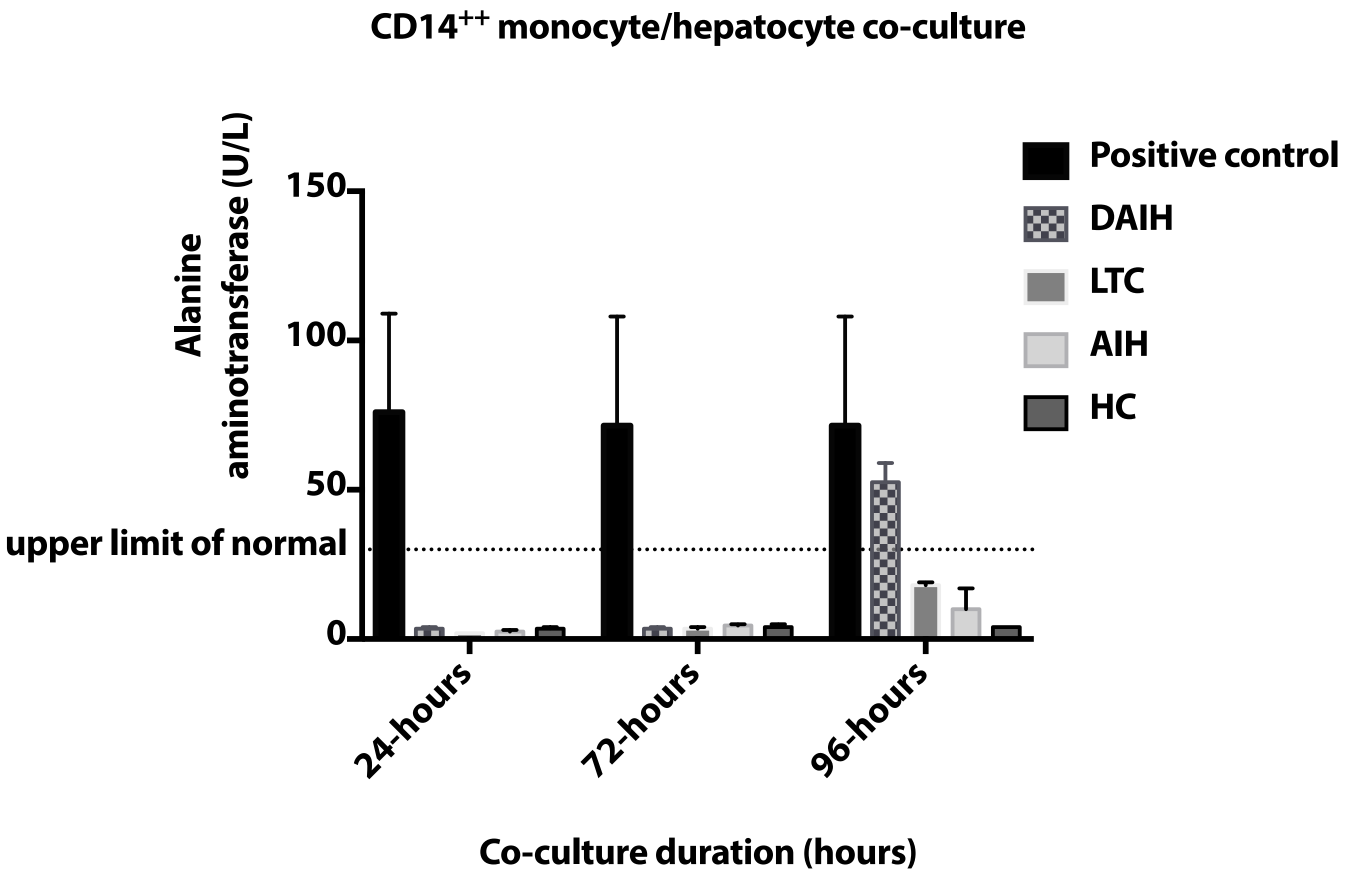
CD14++ monocyte/hepatocyte co-culture
Alanine aminotransferase (U/L)
150
100
50
0
upper limit of normal
24-hours
72-hours
96-hours
Co-culture duration (hours)
Positive control
DAIH
LTC
AIH
HC

SUPPLEMENTARY TABLE 1: Single cell RNA-sequencing cell numbers.

Subject ID	Absolute cell number	Mean reads per cell
**CUMC009**	410	197,906
**CUMC018**	2147	44,391
**HSCC022**	1181	91,447
**HSCC003**	844	101,943
**HSCC014**	9494	11,188
**DN076**	1830	46,847
**DN083**	126	746,226
**DN083**	739	97,836
**DN079**	4472	19,849

SUPPLEMENTARY TABLE 2: Serum alanine aminotransferase among subject groups.

	Subject group		
	DAIH (N = 11)	***LTC (N = 42)***	***AIH (N = 9)***
**ALT (U/L)**			
Median (Range)	35.0 (16.0 – 55.0)	24.0 (12.0 – 57.0)	52.0 (18.0 – 680.0)

SUPPLEMENTARY TABLE 3: Correlation between DAMPs and Alanine aminotransferase in subjects with DAIH.

Spearman Correlation Coefficients		
**DAMP**	Correlation Coefficient	p value
**qPCR ACTB**	0.03	0.95
**qPCR HMGB1**	-0.66	0.15
**qPCR MTATP6**	-0.14	0.78
**qPCR HSP60**	0.10	0.87
**qPCR HSP70**	0.30	0.62
**qPCR HSP90**	-0.30	0.62
**qPCR IL-1β**	0.49	0.32
**qPCR Fibrinogen**	-0.10	0.87
**HMGB1 (pg/ml)**	0.43	0.39
**HSP60 (ng/ml)**	-0.60	0.40
**HSP70 (ng/ml)**	-0.60	0.40
**HSP90 (ng/ml)**	-0.60	0.40
**MtDNA Complex I (ng/ml)**	-0.80	0.20
**MtDNA Complex II (ng/ml)**	-0.80	0.20
**MtDNA Complex III (ng/ml)**	-0.60	0.40
**MtDNA Complex IV (ng/ml)**	-0.80	0.20
**MtDNA Complex V (ng/ml)**	-0.60	0.40

SUPPLEMENTARY TABLE 4: Correlation between DAMPs and Alanine aminotransferase in subjects with AIH.

Spearman Correlation Coefficients		
**DAMP**	Correlation Coefficient	p value
**qPCR - ACTB**	0.083	0.83
**qPCR – HMGB1**	-0.317	0.40
**qPCR – MT-ATP6**	-0.733	0.02
**qPCR – HSP60**	-0.417	0.26
**qPCR – HSP70**	-0.467	0.20
**qPCR – HSP90**	-0.200	0.60
**qPCR - Fibrinogen**	-0.283	0.46
**ELISA – HMGB1**	-0.025	0.94
